# Paper-based lead-free thin film X-ray detectors with high sensitivity and superior environmental stability

**DOI:** 10.1038/s41528-026-00607-8

**Published:** 2026-06-13

**Authors:** Qinghua Zhao, Yi Liu, Yingying Chen, Wenjie Gong, Xing Li, Boru Zhou, He Huang, Ning Han, Shuyu Chen, Baoqiang Zhang, Jian Li, Andres Castellanos-Gomez, Volodymyr Gnatyuk, Weina Nan, Tao Wang

**Affiliations:** 1 School of Materials Science and Engineering, Northwestern Polytechnical University, Xi’an, PR China; 2 State Key Laboratory of Solidification Processing, Northwestern Polytechnical University, Xi’an, PR China; 3 Key Laboratory of Radiation Detection Materials and Devices, Ministry of Industry and Information Technology, Xi’an, PR China; 4 Tianjin Key Laboratory of Functional Crystal Materials, Institute of Functional Crystals, School of Materials Science and Engineering, Tianjin University of Technology, Tianjin, PR China; 5 State Key Laboratory of Crystal Materials, Tianjin Key Laboratory of Functional Crystal Materials, Institute of Functional Crystals, Tianjin University of Technology, Tianjin, PR China; 6 Digital Publishing Department, Shaanxi Science and Technology Press, Xi’an, PR China; 7 Chongqing Jiana Instruments Co Ltd, Chongqing, PR China; 8 Shaanxi Imdetek Co.Shaanxi Imdetek Co. Ltd, Xi’an, PR China; 9 2D Foundry research group. Instituto de Ciencia de Materiales de Madrid (ICMM-CSIC), Madrid, Spain; 10 Advafab Oy, Helsinki, Finland

**Keywords:** Engineering, Materials science, Nanoscience and technology, Physics

## Abstract

Low-dimensional, lead-free halide perovskites are promising candidates for next-generation radiation detectors due to their favorable optoelectronic properties and environmental safety. Among them, Cs_3_Bi_2_Br_9_ has thus attracted significant attention for its layered structure and intrinsic stability. Although Cs_3_Bi_2_Br_9_ has already been explored for X-ray detection, simple and scalable fabrication routes for its thin films on flexible low-cost substrates remain underdeveloped. Here, we demonstrate a sustainable, all-dry abrasion method to directly deposit Cs_3_Bi_2_Br_9_ thin films onto biodegradable office-grade printing paper at room temperature, enabling the fabrication of flexible, lead-free X-ray detectors. The resulting devices exhibit a resistivity of 6.9 × 10^9^ Ω cm and a mobility-lifetime (*μτ*) product of 9.07 × 10−^4^ cm² V⁻¹, yielding X-ray detection sensitivities of 660–860 μC Gy_air_⁻¹ cm⁻². Reducing the electrode spacing to 50 μm further enhances sensitivity to ~28,000 μC Gy_air_^−1^ cm^−2^ which is one of the largest values that reported for thin film-based X-ray detectors, indicating strong potential for miniaturized applications. In addition to high sensitivity, the devices deliver effective imaging performance with a spatial resolution of 1.0 lp/mm (with 28 cm between X-ray source and detectors) and retain stable operation after more than 30 days of air exposure. This work introduces a green and scalable platform for developing high-performance, lead-free, and environmentally sustainable X-ray detectors.

## Introduction

Ionizing radiation, including X-rays and γ-rays, has been widely used in the field of medical imaging, security screening, industrial non-destructive testing, environmental monitoring and scientific research^1–3^. Radiation detection, which converts high-energy photons into electrical signals, is central to these applications and has been extensively studied for over a century. A range of semiconductor materials, based on direct detection principles mechanism, such as silicon (Si)^4^, amorphous selenium (*α*-Se)^5^, mercuric iodide (HgI₂)^6^, cadmium telluride (CdTe)^7^, and cadmium zinc telluride (CdZnTe)^8,9^, have been developed and widely implemented. However, despite their success, each of these materials faces inherent limitations that restrict further advancement in radiation detection. Silicon, for example, suffers from low X-ray absorption, making it suitable only for soft X-rays below 10 keV^10^. *α*-Se is highly sensitive to environmental conditions and undergoes crystallization above 65 °C, which limits its thermal stability^11,12^. CdTe and CdZnTe detectors, especially spectroscopic-grade single crystals, can be costly and brittle and involve cadmium toxicity^13^.

In recent years, halide perovskites with the general formula ABX₃ (where A = CH₃NH₃⁺ (MA⁺), HC(NH₂)₂⁺ (FA⁺), or Cs⁺; B = Pb²⁺ or Sn²⁺; and X = Cl⁻, Br⁻, or I⁻) have attracted significant attention for ionizing radiation detection thanks to their exceptional optoelectronic properties^1,2^. Several key advantages, including high X-ray absorption coefficients, high resistivity, efficient charge transport, intrinsic defect tolerance, and the potential for low-cost crystal growth have been reported on halide perovskites^1,2,14^. Since the first melt-grown metal halide perovskites were reported in 2013^15^, numerous perovskite-based detectors with competitive performance metrics have been demonstrated. For instance, a record-high carrier mobility-lifetime product of 1.2 × 10⁻² cm² V⁻¹ and an exceptionally low surface recombination velocity of 64 cm s⁻¹ based on methylammonium lead bromide (MAPbBr₃) single crystals was obtained^16^. An ultralow limit of detection (LoD) of 0.22 nGyₐᵢᵣ s⁻¹ using MAPbI_3_ was achieved^17^. A record high sensitivity of 2.2 × 10^8^ μC Gyₐᵢᵣ^−1^ cm^−2^ was reported for 8 keV photons at dose rates below 1 μGy s^−1^ utilizing trap-induced photoconducting gain^18^. More recently, an energy resolution of 0.7% at 662 keV using defect-repaired FAPbBr_3_ detectors, highlighting the potential of halide perovskites for γ-ray detection, was reported^19^.

Despite remarkable progress in perovskite-based radiation detectors, two critical challenges continue to hinder their practical deployment: limited operational stability and the presence of toxic lead. Low-dimensional, lead-free perovskites, particularly Cs₃Bi₂Br₉, offer a promising path forward. This bismuth-based compound not only complies the Restriction of Hazardous Substances (RoHS) regulations and simultaneously exhibits strong radiation detection performance due to several key material attributes. First, Cs₃Bi₂Br₉ offers strong X-ray attenuation, enabled by its relatively high average atomic number (Z = 46.1) and density (4.7 g cm^−3^), surpassing *α*-Se (Z = 34)^5^ and MAPbBr₃ (Z = 41.2)^16^, and Cs₂AgBiBr₆ (*Z* = 45)^20^, and approaching iodide-based materials such as (NH₄)₃Bi₂I₉ (Z = 48.3)^21,22^. Second, Cs₃Bi₂Br₉ possesses a wide band gap of 2.61 eV, which can greatly suppresses thermal carrier generation and enables ultralow dark currents (17.8 pA mm^−1^ at 100 V), while its high resistivity (1.79 × 10¹¹ Ω cm) ensures low noise floor^23,24^. Third, Bi³⁺ ions share a similar electronic configuration (6 s²6p⁰) with Pb²⁺, leading to favorable optoelectronic properties through comparable band structures and spin–orbit coupling. Devices based on Cs_3_Bi_2_Br_9_ have demonstrated a high mobility-lifetime product of 8.32 × 10⁻⁴ cm² V⁻¹, a sensitivity of 7304 μC Gy⁻¹ cm⁻², and a detection limit as low as 0.58 nGy s⁻¹^25^, comparable to the early-stage CdTe detectors^8^. More importantly, the material’s structural stability is further reinforced by its two-dimensional layered architecture. A high formation energy (1.8 eV atom^−1^) provides resistance to phase decomposition, while van der Waals gaps between [Bi₂Br₉]³⁻ layers eliminate dangling bonds, reducing susceptibility to ambient degradation^26,27^. Furthermore, a diffusion barrier of 0.7 eV—more than three times higher than that of conventional 3D perovskites—effectively suppresses ion migration, supporting long-term electrical stability^28^.

Despite these abovementioned advantages, translating the material potential of Cs_3_Bi_2_Br_9_ into scalable devices remains challenging. While bulk single crystals offer excellent performance, they are costly to produce and limited in size. In contrast, thin-film fabrication—crucial for large-area and flexible X-ray detectors—remains largely underdeveloped due to unfavorable crystallization dynamics and thermal sensitivity.

Perovskite film fabrication techniques are broadly classified into solution-based deposition and solvent-free approaches^29^, each posing distinct challenges for the scalable development of Cs_3_Bi_2_Br_9_-based radiation detectors. Solution-based techniques, such as spin-coating^30^, drop-casting^31,32^, and inkjet printing^33^, rely on dissolving precursor salts in volatile solvents to form printable inks. Although these methods enable room-temperature processing, they are limited by the poor solubility of Cs_3_Bi_2_Br_9_ precursors due to strong ionic bonding. Incomplete dissolution often results in non-uniform crystallization and unreacted residues. Furthermore, rapid solvent evaporation during deposition leads to porous films with uncontrolled grain boundaries, which is particularly detrimental for radiation detection, where dense, defect-free active layers are essential. Solvent-free methods—including hot pressing and physical vapor deposition techniques such as sputtering and closed-space sublimation—bypass solvent-related issues by consolidating pre-synthesized perovskite powders^34–36^. While these vacuum-based processes can yield compact crystalline films, they require specialized equipment and are energy-intensive, often involving high-temperature processing (above 400 °C)^34^. Additionally, the intrinsic brittleness of Cs_3_Bi_2_Br_9_ crystals complicates mechanical compression, frequently resulting in microcracks that degrade device performance. These technical and economic barriers present significant obstacles to scaling current fabrication strategies for producing robust, large-area Cs_3_Bi_2_Br_9_ membranes suitable for practical radiation detection applications. While previous studies have demonstrated the feasibility of Cs_3_Bi_2_Br_9_ thin films for X-ray detection^37^, practical fabrication routes that are simple, solvent-free, and compatible with flexible low-cost substrates remain underexplored.

In this work, we introduce an all-dry abrasion deposition strategy for directly forming functional Cs_3_Bi_2_Br_9_ films on biodegradable printing paper at room temperature. As a substrate, printing paper offers several advantages—low cost, flexibility, and biodegradability—making it well-suited for the development of eco-friendly and scalable radiation detection systems. The layered structure of Cs_3_Bi_2_Br_9_ enables the formation of uniform semiconducting films, tens of micrometers thick, with strong adhesion and good homogeneity across the paper surface. Electrical characterization reveals that the fabricated films exhibit a resistivity of 6.9 × 10^9^ Ω cm and a mobility-lifetime (*μτ*) product of 9.07 × 10^−4 ^cm² V⁻¹, yielding X-ray detection sensitivities of 660–860 μC Gy_air_⁻¹ cm⁻². Sensitivity is further enhanced to approximately 28,000 μC Gy_air_⁻¹ cm⁻² by reducing the electrode spacing down to 50 μm, demonstrating strong potential for miniaturized applications. Notably, the devices maintain stable performance after more than 30 days of ambient air exposure, confirming their excellent environmental and operational durability. We further construct a single-pixel X-ray imaging system based on these paper-supported detectors, illustrating their feasibility for low-cost, flexible, and high-performance radiation sensing. This work offers a sustainable and potentially scalable platform for flexible lead-free radiation detectors.

## Results

### Cs_3_Bi_2_Br_9_ bulks and powders

**Fig. 1:**
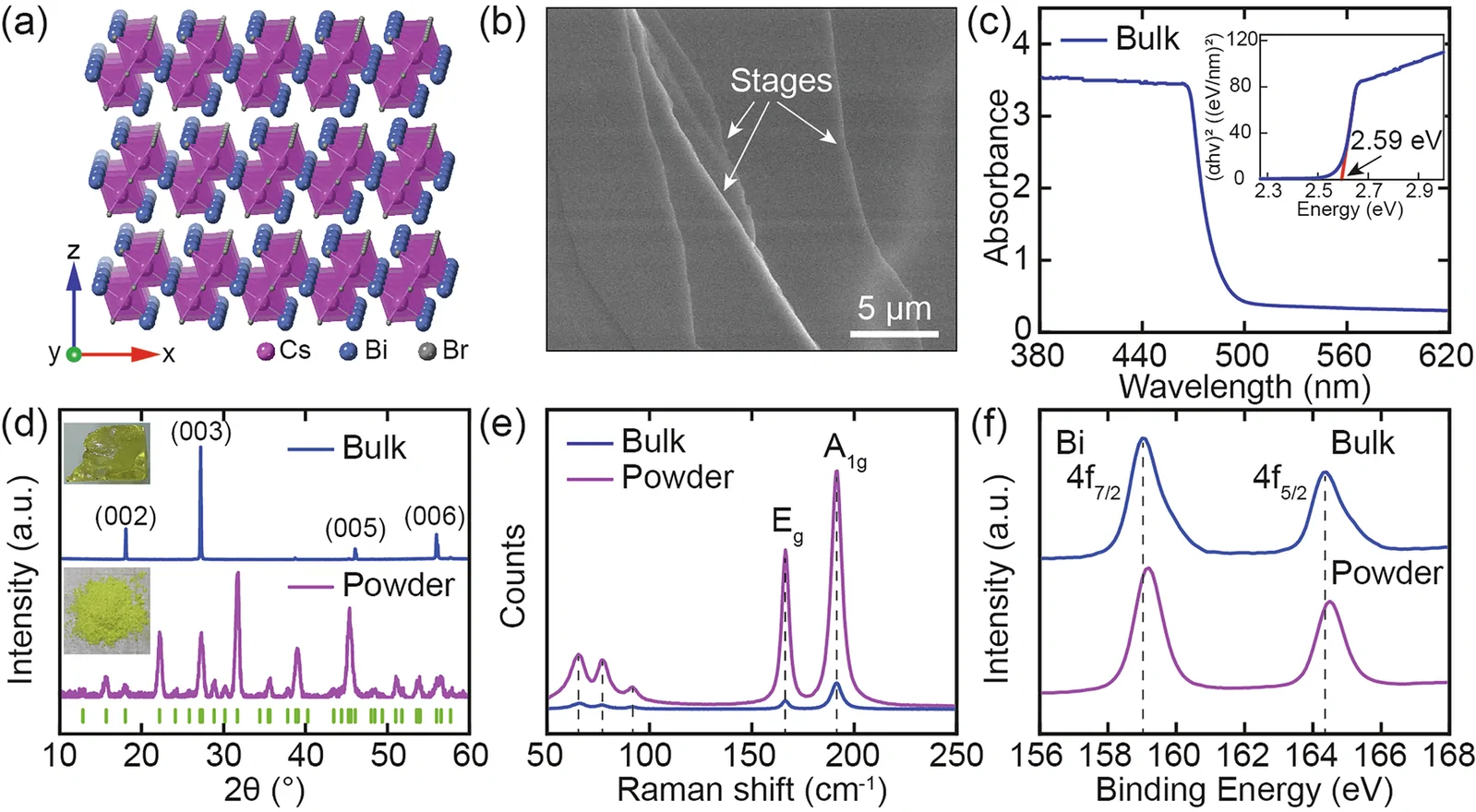
Layered semiconducting Cs_3_Bi_2_Br_9_ bulks and powders. **a** Lattice structure of Cs_3_Bi_2_Br_9_. The Cs, Bi, and Br atoms are represented in different colors. **b** Scanning electron microscopy (SEM) image showing the cleavage plane stages of Cs_3_Bi_2_Br_9_ crystals, the optical contrast of the image has been enhanced for better visualization of the stages. **c** Absorption spectra of Cs_3_Bi_2_Br_9_ single crystals. Inset: calculated Tauc plot with a direct band gap of 2.59 eV. Characterizations of Bi in Cs_3_Bi_2_Br_9_ single crystals and powders after grinding: **d** X-ray diffraction (XRD), **e** Raman spectroscopy, and **f** X-ray photoelectron spectroscopy (XPS).

Cs_3_Bi_2_Br_9_ is an all-inorganic perovskite with a hexagonal crystal structure, characterized by lattice parameters of *a* = *b* = 7.978 Å, *c* = 9.852 Å, and *α* = *β* = 90°, *γ* = 120°^38^. Figure 1a depicts the lattice structure of Cs_3_Bi_2_Br_9_, highlighting the atomic planes bound by van der Waals forces. The layered nature, consisting of corner-sharing [BiBr_6_]³⁻ octahedra surrounded by Cs cations, allows for easy exfoliation of Cs_3_Bi_2_Br_9_ single crystals along the (001) plane. The smooth surface with few steps is clearly visible in the scanning electron microscope (SEM) image in Fig. 1b. The stoichiometric ratio of Cs_3_Bi_2_Br_9_ was confirmed using energy-dispersive spectroscopy (EDS) characterization, with results provided in Fig. S1 (see Supporting Information). Further optical property characterizations were conducted on these perovskite bulk materials, and the absorbance variation of Cs_3_Bi_2_Br_9_ crystals across incident wavelengths from 380 to 620 nm is shown in Fig. 1c, where a sharp absorption edge at approximately 480 nm is evident. The inset indicates that the bandgap was investigated using a Tauc plot, determining a direct bandgap of 2.59 eV for Cs_3_Bi_2_Br_9_, which is consistent with previously reported results^24,39^.

After confirming the high crystallinity of the bulk crystals, we proceeded to characterize the Cs_3_Bi_2_Br_9_ perovskite powders. Figure 1d compares the X-ray diffraction (XRD) spectra of Cs_3_Bi_2_Br_9_ crystals before and after being ground into powders. The bulk XRD plot reveals four sharp diffraction peaks at 18.06°, 27.20°, 46.05°, and 55.98°, corresponding to the (002), (003), (005), and (006) cleavage planes, respectively. This indicates that the bulk crystals exhibit a well-oriented single-crystal structure along the *c*-axis. In the powder XRD plot, a good match is observed between the experimental data and the standard X-ray diffraction pattern of Cs_3_Bi_2_Br_9_ (PDF #44-0714). Peak broadening is observed in the powder relative to the bulk crystal (Quantitative FWHM comparisons for identical reflections are provided in Table S1), which can be explained by the reduced coherent domain size (1–5 μm range in Fig. S2) and increased macrostrain induced by mechanical processing^40^.

Figure 1e presents the Raman spectrum acquired from Cs_3_Bi_2_Br_9_ bulk and powder samples, both exhibiting similar profiles with five prominent peaks at 65.4 cm⁻¹, 76.9 cm⁻¹, 91.6 cm⁻¹, 166.4 cm⁻¹, and 191.2 cm⁻¹. These peaks can generally be divided into two groups: the low-frequency Raman shifts at 65.4 cm⁻¹, 76.9 cm⁻¹, and 91.6 cm⁻¹, which may originate from the deformation vibrations of the Bi-Br bond, translational modes of Cs⁺, and the stretching modes of the BrBi_2_ groups^41^; and the high-frequency Raman shifts at 166.4 cm⁻¹ and 191.2 cm⁻¹, which are due to the in-plane and out-of-plane vibrational modes of the [BiBr_6_]³⁻ octahedron, corresponding to the E_g_ and A_1g_ modes, respectively^41,42^. Notably, the Raman signal intensity of the Cs_3_Bi_2_Br_9_ powder is significantly stronger approximately 9 times that of the bulk crystals. This enhancement may be attributed to the resonance of the optical cavity caused by photon confinement effects in the perovskite powder^43^.

Given the environmental stability of the perovskite crystals^44^, we further investigated the elemental composition and chemical valence of the Cs_3_Bi_2_Br_9_ powders using X-ray photoelectron spectroscopy (XPS). The XPS spectrum (Fig. S3) confirms the presence of Cs, Bi, and Br in Cs_3_Bi_2_Br_9_. As shown in Fig. 1f, after the Cs_3_Bi_2_Br_9_ bulk single crystal was ground into powder, the peak positions of Bi 4f_7/2_ and 4f_5/2_ shifted from 158.75 eV and 164.15 eV to 158.9 eV and 164.3 eV, respectively, corresponding to a blue shift of 0.15 eV for both components. Owing to the large surface area and abundant defect sites in the Cs_3_Bi_2_Br_9_ powders, there are more oxygen and water species can be adsorbed on the powder surface, thus introducing Bi–O formation effects during ambient handling. These surface modifications seize electrons from Bi atoms, further increasing the Bi 4 f binding energy and producing the observed blue shift^45^.

### Fabrication and characterization of paper-based Cs_3_Bi_2_Br_9_ films

**Fig. 2:**
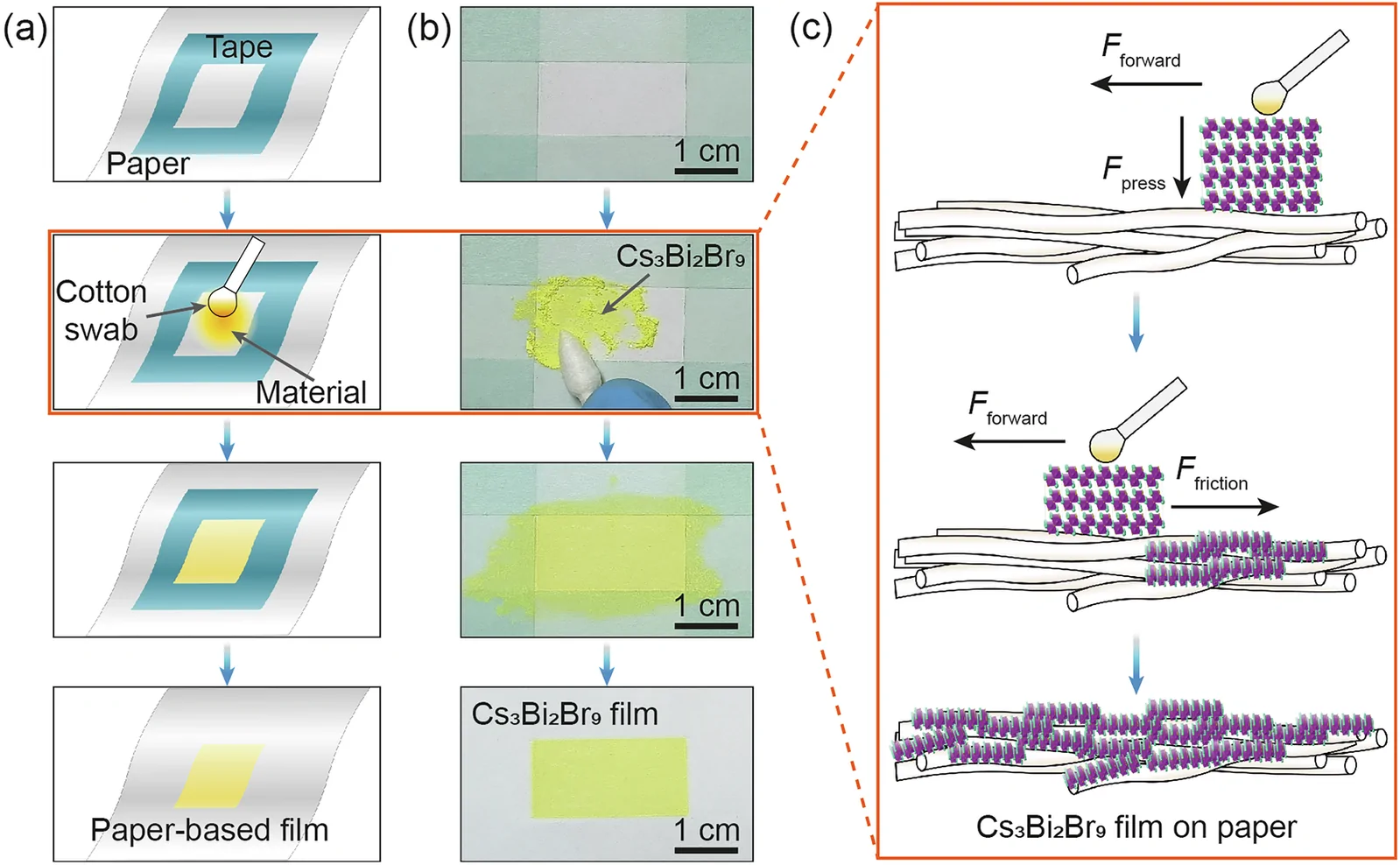
Fabrication of paper-based Cs_3_Bi_2_Br_9_ films. **a** Schematic illustration of the fabrication process for paper-based Cs_3_Bi_2_Br_9_ films using an all-dry abrasion deposition technique. **b** Photographs showing different stages of Cs_3_Bi_2_Br_9_ film preparation. **c** Schematic diagrams illustrating the material deposition process during abrasion.

After verifying the elemental purity and crystallinity of the Cs_3_Bi_2_Br_9_ powders, we utilized them to fabricate paper-based perovskite thin films. As illustrated in Fig. 2a (cartoon illustration) and Fig. 2b (photograph of the actual process), adhesive tape (paper masking tape) was first used to create a mask of rectangular areas on standard printing paper (70 g m^−2^). The Cs_3_Bi_2_Br_9_ powders were then rubbed onto the exposed area using a cotton swab. As the perovskite material spread along the paper surface, a homogeneous and continuous Cs_3_Bi_2_Br_9_ film began to form. Afterward, the edge-limiting masking tape was removed, leading on the paper to a rectangular shaped film. This fabrication methodology is commonly referred to as all-dry abrasion deposition^46,47^, a technique widely employed in paper-based electronics^48–51^.

To better understand the deposition mechanism, a detailed schematic is provided in Fig. 2c. During the pressing and abrasion process with the powder-coated cotton swab, the interlayer van der Waals bonds within the Cs_3_Bi_2_Br_9_ microcrystals are broken by the friction between the rough paper surface and the perovskite materials, resulting in crystal cleavage and subsequent deposition on the paper surface. As more crystals accumulate, inter-crystal friction gradually replaces the friction between the crystals and the paper. When displacement rather than cleavage occurs between microcrystals, the film reaches its maximum thickness, and deposition ceases. Due to the deposition of Cs_3_Bi_2_Br_9_ material not only on the surface of the cellulose fibers but also within the gaps between them, variations in the thickness of the prepared films are observed. The material layer deposited on the fibers is denser and thinner compared to the layer within the gaps.

**Fig. 3:**
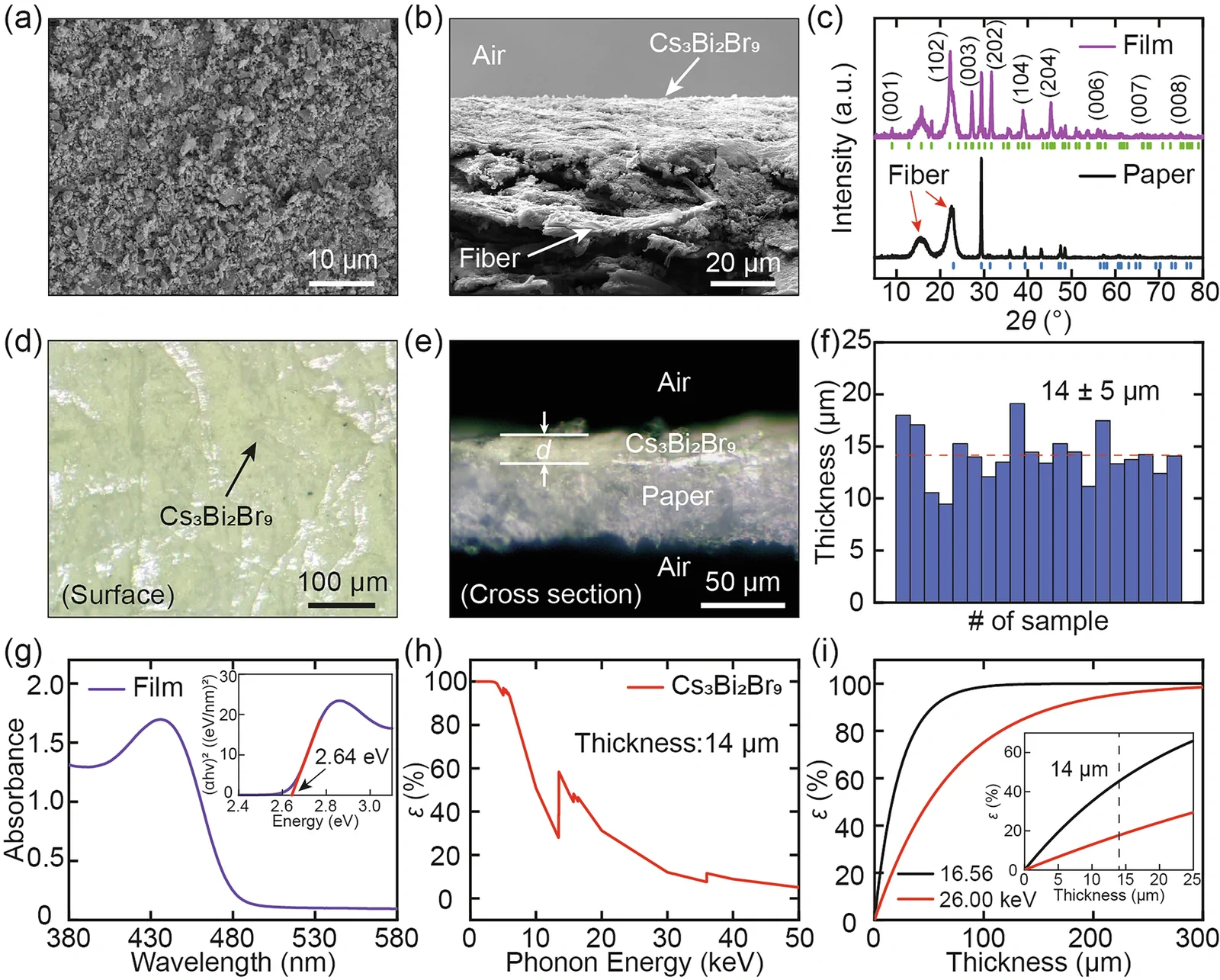
Characterizations of paper-based Cs_3_Bi_2_Br_9_ perovskite films. Scanning electron microscopy (SEM) images of the surface (**a**) and cross-section (**b**) of paper-based Cs_3_Bi_2_Br_9_ films. **c** X-ray diffraction (XRD) patterns of paper-based Cs_3_Bi_2_Br_9_ films (purple) and the paper substrate (black). **d**, **e** Optical micrographs of the Cs_3_Bi_2_Br_9_ film surface and cross-section; the thickness is measured in (**e**). **f** Histogram of thickness measurements from 20 cross-section locations, estimating the statistical thickness distribution of the films, with an average thickness of ~14 μm. **g** Absorbance variation as a function of incident wavelength for paper-based Cs_3_Bi_2_Br_9_ films. Inset: calculated Tauc plot, indicating a direct band gap of 2.64 eV. **h** Attenuation coefficient as a function of incident photon energy (1–50 keV) calculated for a 14 μm-thick Cs_3_Bi_2_Br_9_ film. **i** Calculated relationship between attenuation coefficient and Cs_3_Bi_2_Br_9_ film thickness at X-ray tube voltages of 20 kVp (black) and 40 kVp (red), respectively. Inset: zoomed-in view for small thicknesses.

Compared to methods such as spray-coating^52^, spin coating^53^, and dip coating^54^, the abrasion method is not only a simple, fast, and environmentally friendly strategy but also avoids contamination from organic solvents that tend to accumulate at the flake-to-flake interfaces hampering a good electrical conductivity. To gain more detailed insights into the morphology and composition of the films prepared by this method, we conducted basic observations and characterizations on paper-based perovskite films. The films were initially examined using a high-resolution scanning electron microscope (SEM) and an optical microscope. As seen in the SEM image in Fig. 3a, the Cs_3_Bi_2_Br_9_ powder completely fills the micropores formed by intersecting fibers, with only a few particles remaining loosely on the surface. Additionally, adherence of the microcrystals to the fibers is observable from the cross-sectional view (Fig. 3b), confirming the formation of a relatively flat surface.

In Fig. 3c, the X-ray diffraction (XRD) results of the film show distinctive diffraction peaks of Cs_3_Bi_2_Br_9_, along with peaks identified as cellulose^55^ (diffuse diffraction peaks at 15.6°, 22.6°) and calcium carbonate fillers (blue lines). Optical microscopy (Fig. 3d) reveals that Cs_3_Bi_2_Br_9_ forms a relatively continuous film on the paper surface, an observation further supported by the energy-dispersive spectroscopy (EDS) analysis in Fig. S5. Cross-sectional optical microscopy imaging was then performed to evaluate the thickness of the Cs_3_Bi_2_Br_9_ film (Fig. 3e). Considering the variation in the thickness of the paper-based film, statistical measurements were taken from 20 different samples, yielding an average thickness of approximately 14 μm, with a variation of about ±5 μm (Fig. 3f). This aligns with energy-dispersive X-ray spectroscopy (EDS) elemental mapping, which estimated a comparable thickness of ~16 μm (Fig. S6). The observed nonuniformity in the film closely mirrors the topography of the original printing paper. This indicates that the Cs_3_Bi_2_Br_9_ material deposits conformally along the substrate’s natural fibrous texture, indicating further optimization is required to improve film uniformity in future work.

The absorption spectrum of the Cs_3_Bi_2_Br_9_ film, shown in Fig. 3g, reveals a direct bandgap (*E*_g_) of 2.64 eV as determined from the Tauc plot. This value is 0.05 eV larger than that of the bulk crystals, which can be attributed to a slight quantum confinement effect induced by the reduced size^56^. These results demonstrate that the Cs_3_Bi_2_Br_9_ powder can adhere securely to paper surfaces, forming a continuous and well-packed films composed of interconnected flakes.

For enhanced use in X-ray detection, a deeper understanding of the X-ray cut-off capabilities of thin films is essential. Based on the obtained parameters, we calculated the linear absorption coefficient (*μ*) of Cs_3_Bi_2_Br_9_ in the photon energy range of 1–50 keV using the photon cross-section database^57^. The attenuation coefficient (*ɛ*) of the film was then determined from *μ* using the following equation:

1 $$\varepsilon =1-{e}^{-\mu x}$$

where *μ* is the linear absorption coefficient and *x* is the film thickness, which is 14 μm in this case. As shown in Fig. 3h, the attenuation coefficient of the film reaches a peak value at a photon energy of approximately 13 keV and then decreases as the energy increases. Additionally, when the tube voltage is fixed, the relationship between the attenuation coefficient and film thickness can be further evaluated. Under the 20 and 40 kVp spectra (approximated by spectrum-averaged energies of 16.56 and 26.00 keV, respectively in Fig. S7), the film is estimated to attenuate ~46% and ~18% of incident X-rays, respectively, as depicted in Fig. 3i. This absorption level is comparable to that reported for CsPbBr3 thin films^58^, and supports the feasibility of using abrasion-deposited Cs3Bi2Br9 films for flexible X-ray detection, while also highlighting the need for further optimization of film thickness and compactness.

To further investigate the X-ray detection performance of the Cs_3_Bi_2_Br_9_ film, Au- Cs_3_Bi_2_Br_9_-Au X-ray detectors with a coplanar structure were fabricated by sputtering interdigitated gold electrodes onto the thin films using a shadow mask. The device features coplanar Au electrodes patterned onto the paper substrate in an interdigitated configuration, whose geometrical illustration has been shown in Fig. S8 and Table S2.

### X-ray detection performance of paper-based Cs3Bi2Br9 detectors

**Fig. 4:**
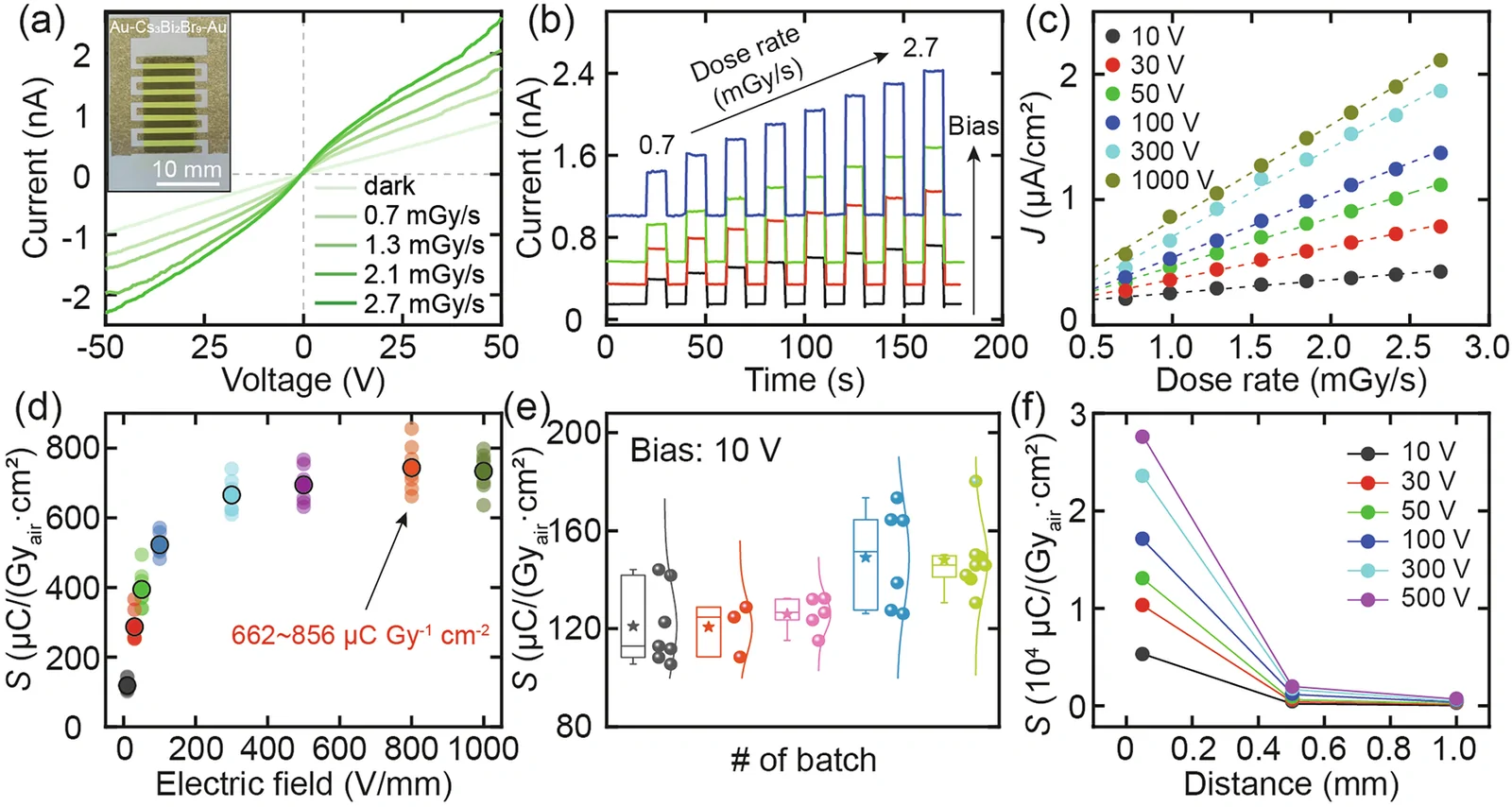
X-ray detection performance of paper-based Cs_3_Bi_2_Br_9_ detectors. **a** Current-voltage (*I–V*) curves recorded under dark conditions and under illumination with varying X-ray doses. **b** Current at *V* = 10 V (black), 30 V (red), 50 V (green), and 100 V (blue) recorded by a Cs_3_Bi_2_Br_9_ detector as a function of time while turning a 40 keV X-ray source on and off, with radiation dose rates ranging from 0.7 mGy s^−1^ to 2.7 mGy s^−1^. **c** Photocurrent density as a function of X-ray illumination dose rate recorded at six different bias voltages. **d** Variation of X-ray detection sensitivity (*S*) plotted as a function of the applied electric field. Small circles represent individual data points, and large circles represent the average values for each group. **e** Statistical distribution of detection sensitivity (*S*) in Cs_3_Bi_2_Br_9_ devices prepared in different batches at a 10 V bias voltage. The sensitivity values from each batch are fitted to a Gaussian distribution, with stars indicating the extracted average values and squares indicating the 25–75% data concentration range. **f** Relationship between sensitivity and channel distance at different bias voltages.

Figure 4a presents the current-voltage (*I–V*) curves of a Cs_3_Bi_2_Br_9_ detector (the width and pitch of the interdigitated electrode are both 1 mm), measured in the dark and under different X-ray dose rates. From the dark branch, we determined a high resistivity of 6.9 × 10^9^ Ω cm for our device (representative dark *I–V* curve and statistical analysis can be referred to Fig. S9). As the X-ray dose rate increases, more electron-hole pairs are generated and separated, resulting in an increased carrier flow within the devices, which leads to higher current and lower resistance. As shown in Fig. S10, the voltage-dependent X-ray photoconductivity was fitted using the modified Hecht equation, yielding an effective *μτ* product of approximately 10^-4 ^cm^2 ^V^−1^ for the paper-based Cs3Bi2Br9 detector. This value is higher than that of Cs_2_TeI_6_ (5.2 × 10⁻⁵ cm² V⁻¹)^59^, MA_3_Bi_2_I_9_ (3.89 × 10⁻⁵ cm² V⁻¹)^60^, and MAPbI_3_ (1.0 × 10⁻⁴ cm² V⁻¹)^61^ and is comparable to that of MDABCO-NH₄I₃ (1.64 × 10⁻⁴ cm² V⁻¹)^62^ (a detailed comparison has been presented in the Table S3 in the Supporting Information). The discrepancies among resistivity, *μτ* and sensitivity between our paper-based devices and bulk crystals also have been discussed in supporting information (after Fig. S10).

Figure 4b illustrates the time response characteristics, where the current flowing through the device is recorded as a function of time while turning the X-ray irradiation (with a tube voltage of 40 kV_p_) ON and OFF at different bias voltages. At a fixed bias, the current in the device shows a linearly increasing trend as the X-ray dose varies from 700 to 2700 μGy_air_ s^−1^. When the applied bias voltage is gradually increased, the current in the device under both dark conditions (*J*_dark_) and X-ray illumination (*J*) also increases, resulting in a net increase in photocurrent (*J*–*J*_dark_). To evaluate the sensitivity of our devices, these photocurrent values are plotted as a function of X-ray dose in Fig. 4c. The sensitivity of the coplanar X-ray detector was calculated using the following equation^18,63^:

2 $$S=\frac{{J}_{\mathrm{light}}-{J}_{\mathrm{dark}}}{D}$$

Where *J*_light_ (*J*_dark_) are the current densities under illumination and in the dark, respectively, and *D* is the X-ray dose rate in air. For the coplanar device geometry, the current density was obtained by dividing the measured current by the effective conduction cross-sectional area, ${A}_{\mathrm{eff}}=w\times h$, where *w* is the channel width and *h* is the film thickness.

The photocurrent and dose rate extracted at various bias voltages were fitted to a linear relationship, allowing us to determine the device sensitivity by examining the slope values. Figure 4d illustrates how the X-ray detection sensitivities of our devices vary as a function of operating voltage. Four different device samples exhibit a consistent variation pattern: under low voltage conditions, the sensitivity of the device gradually increases as the voltage rises. However, at higher voltage levels, the rate of sensitivity growth slows down until it reaches saturation. This phenomenon can be explained by the increased carrier collection efficiency with a stronger electric field^64^. Notably, the plot shows that our paper-based Cs_3_Bi_2_Br_9_ detectors can achieve a maximum sensitivity of 661.7–855.9 μC Gy⁻¹ cm⁻² at 800 V, a value that can be explained by trap-induced photoconductive gain effect^37^, which is significantly higher than that of lead-free thin-film X-ray detectors, such as Cs_3_Bi_2_I_9_ (127.23 μC Gy⁻¹ cm⁻²)^65^, Cs_2_TeI_6_ (76.27 μC Gy⁻¹ cm⁻²)^66^, Cs_2_AgBiBr_6_ (40 μC Gy⁻¹ cm⁻²)^67^, and (BA)₂Cs_9_Ag_5_Bi_5_Br_31_ (46.98 μC Gy⁻¹ cm⁻²)^68^, and approximately 30 times larger than that of *α*-Se detectors^11^.

To further assess the reproducibility of our fabrication method and device performance, we conducted a statistical analysis based on devices prepared in five different batches. As shown in Fig. 4e, the sensitivity at a 10 V bias is concentrated around the peak of the Gaussian distribution. Sensitivity values across different batches remain within the range of 110–160 μC Gy⁻¹ cm⁻², indicating consistency and good reproducibility. Moreover, we also evaluated the performance stability of our devices over different time scales. Figure S11 explores the operational stability of the paper-based Cs_3_Bi_2_Br_9_ device, showing that the net photocurrent remains essentially unchanged over approximately 100 cycles, although both dark and photocurrents exhibit a slight decrease. In addition, we investigated the long-term stability by monitoring changes in the film’s appearance, optical properties, and device performance over time in an ambient environment. After more than 30 days of exposure to ambient conditions, no significant changes were observed in surface appearance, XRD, photoluminescence (PL), Raman, ultraviolet (UV) absorption, transmission spectra, or detection sensitivity (see Figs. S12 and S13). These results show the Bi oxidation and adsorption of oxygen and water do not cause lasting damage to the detector’s performance, while mitigation of such surface adsorption remains an important direction for future optimization. To evaluates the mechanical durability of the paper-based Cs_3_Bi_2_Br_9_ detectors, we conducted cyclic bending tests with a bending radius of 5 mm for up to 100 cycles. After repeated bending deformation, the devices maintained over 99.6% of their initial photocurrent response, showing negligible performance degradation (Fig. S14). When sharply folded, the detectors still preserved 89.6% of their original sensitivity, and their *I*–*V* and *I*–t responses exhibited no discernible drift (Fig. S15). This environmental stability and mechanical robustness may be attributed to the strong interfacial adhesion between the Cs_3_Bi_2_Br_9_ flakes and the cellulose fiber matrix, as well as the layered structure of Cs_3_Bi_2_Br_9_, which helps slow moisture ingress and accommodates bending without fracture.

Interestingly, we further found that the X-ray detection sensitivity of these paper-based Cs_3_Bi_2_Br_9_ devices can be enhanced by decreasing the channel distance. As shown in Fig. 4f, the maximum sensitivity for a device with a channel distance of 0.5 mm (the electrode width is 1 mm) can reach 1180 μC Gy⁻¹ cm⁻² at 1000 V (see also Fig. S16), which is slightly higher than that of devices with a channel distance of 1.0 mm. However, when the channel distance is reduced to 50 μm, despite a 10-fold reduction in X-ray radiation dose rates, the photocurrent remains comparable to that of previous devices, resulting in a maximum X-ray detection sensitivity of approximately 22,000–30,000 μC Gy^−1^ cm^−2^ at 500 V (see in Figs. S17 and S18). This value is 16 times greater than that of Cs_3_Bi_2_Br_9_ single-crystal detectors (1705 μC Gy⁻¹ cm⁻² for 120 keV X-ray)^25^, surpassing the sensitivity of MA_3_Bi_2_I_9_ (2065 μC Gy⁻¹ cm⁻²)^68^ and some lead-halide perovskites such as MAPbI_3_ (7304 μC Gy⁻¹ cm⁻²)^69^, Cs_0.1_(FA_0.83_MA_0.17_)_0.9_Pb(Br_0.17_I_0.83_)₃ (59.9 μC Gy⁻¹ cm⁻²)^70^, and even comparable to that of quasi-single-crystal CsPbBr₃ film detectors (55,684 μC Gy⁻¹ cm⁻²)^71^. This increase in detection sensitivity induced by reducing the channel distance in our devices may be related to the following factors: (1) the rapid amplification of the electric field increases the kinetic energy of the charges during migration, thereby increasing the carrier diffusion length (*L* = *μτE*), which enables carriers to diffuse over longer distances without being trapped; (2) the porous flake network and defect-rich interfaces introduce the trap-induced photoconductive effect which can temporarily trap and release photocarriers under bias, effectively extending the carrier lifetime relative to the transit time^18,37^; (3) for such a small channel spacing (50 μm), charge cloud produced by the incident radiation near fringing fields can be collected by the electrodes^72^. Taken together, these coupled factors provide an explanation for the higher sensitivity.

**Fig. 5:**
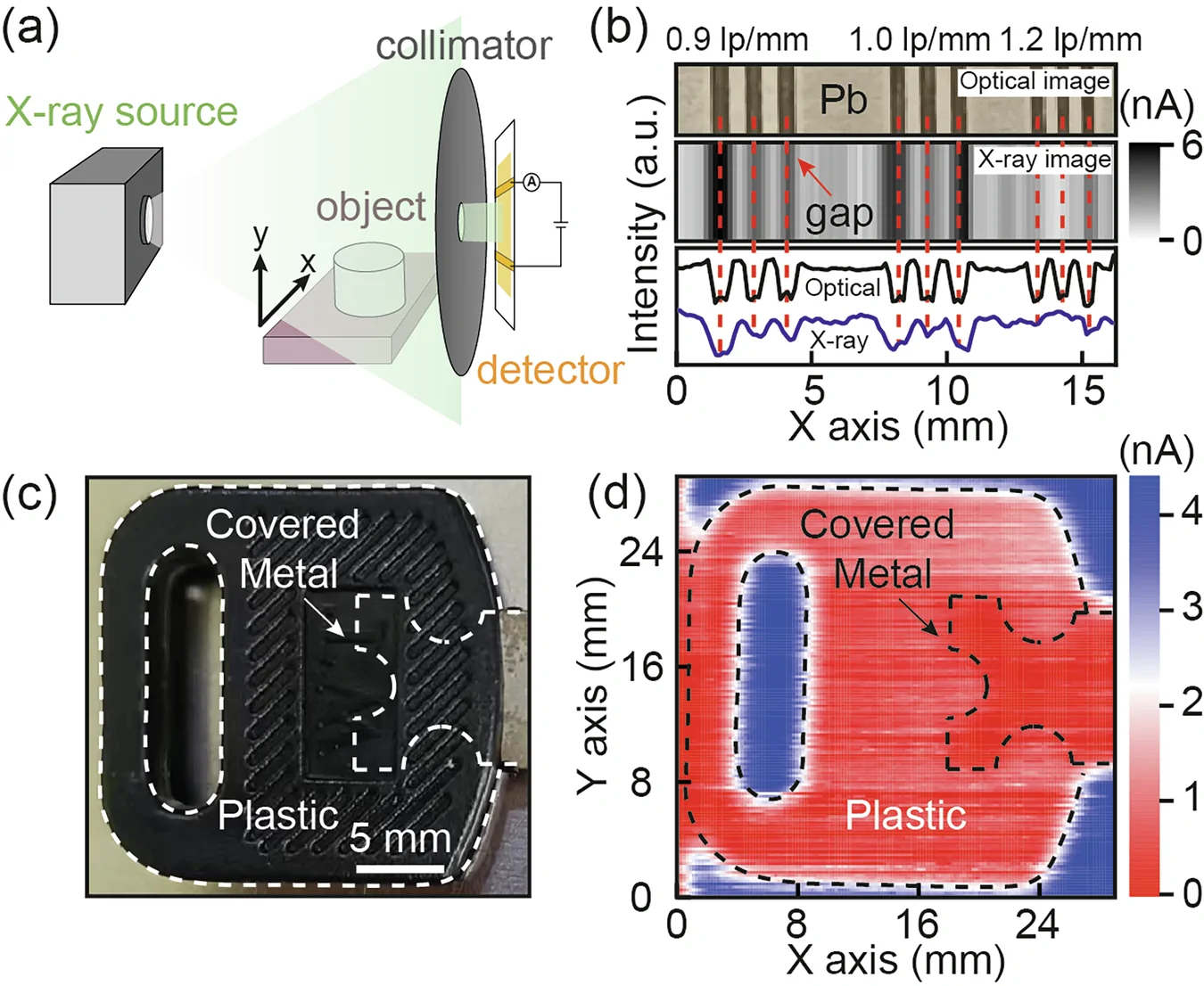
Imaging capability of paper-based Cs_3_Bi_2_Br_9_ detectors. **a** Schematic diagram of X-ray imaging using planar detectors. **b** From top to bottom: optical image, corresponding X-ray image of the standard line card, and a grayscale comparison between them from 0.9 lp mm^−1^ to 1.2 lp mm^−1^. **c** Photograph of the key handle and **d** corresponding X-ray image, with dotted lines marking the external plastic outline and internal metallic components. The color bar on the right represents the extracted photocurrent. Imaging conditions: 20 kVp; measured dose rate at detector plane = 152.8 μGy s^−1^; step size = 0.2 mm; bias = 100 V; exposure time = 0.2 s per step.

The high sensitivity, high resistivity, and superior environmental stability of these paper-based Cs_3_Bi_2_Br_9_ thin-film detectors make them promising candidates for X-ray detection. Their linear response to X-ray illumination further positions them as ideal active elements in imaging applications. To investigate their imaging capability, we employed an x–y point-by-point scanning imaging system. Figure 5a provides a schematic of the experimental setup. Briefly, the object to be imaged is placed on an x–y displacement stage, and a collimator (a lead sheet with a 3 mm diameter hole) transmits parallel X-rays to the detector. During the imaging process, the X-ray source, object, and detector are aligned in parallel with a total travel distance of 28 cm. Variations in the attenuated X-ray dose at different positions, due to differences in material and thickness, result in corresponding variations in the induced photocurrent. By manipulating the displacement platform in the x and y directions, an image is obtained by plotting the final position-correlated current matrix.

In Fig. 5b, the spatial resolution was evaluated using standard line pair X-ray test patterns (Type 81). The results of both optical and X-ray imaging show that the element of 1.0 lp mm⁻¹ can be clearly distinguished by the device with a 50 μm channel. The grayscale curves confirm this, with peaks corresponding to the gaps, indicating a spatial resolution higher than 1.0 lp mm⁻¹. Finally, we conducted a test to confirm the imaging feasibility of our paper-based Cs_3_Bi_2_Br_9_ thin-film detectors. The optical image of a key handle is shown in Fig. 5c, d presents the acquired photocurrent image obtained using an X-ray source with a tube voltage of 20 kVp and a current of 300 μA, raster-scanning the object in the X and Y directions (gray scale and 3D plot can be found in Fig. S19, and another example can be found in Fig. S20). The corresponding plot shows that, in addition to capturing distinct external outlines of the object, it is also possible to observe internal metallic components, further demonstrating the potential applications of paper-based Cs₃Bi₂Br₉ detectors in the X-ray imaging field.

## Discussion

In this work, we successfully developed a thin-film Cs_3_Bi_2_Br_9_ X-ray detector on a paper substrate using an all-dry abrasion methodology. Our results reveal that Cs_3_Bi_2_Br_9_ powders, which inherit the high crystalline quality of their bulk counterparts, can be effectively deposited onto the fibrous surface of the paper due to their layered structural features, forming a continuous and homogeneous thin film. Subsequent investigations show that this paper-based Cs_3_Bi_2_Br_9_ thin film exhibits efficient X-ray absorption capability and holds great promise for X-ray detection applications, as confirmed by the performance of devices with a planar geometry. Electrical characterizations indicate a resistivity of 6.9 × 10^9^ Ω cm and a carrier mobility-lifetime product of 9.07 × 10⁻^4^ cm² V⁻¹ in the fabricated devices, resulting in a high X-ray detection sensitivity of 660–860 μC Gy⁻¹ cm⁻², along with good reproducibility and environmental stability. Interestingly, we further found that the detection sensitivity can be significantly enhanced by reducing the channel distance, achieving a maximum sensitivity of approximately 28,000 μC Gy⁻¹ cm⁻² with a device channel distance of 50 μm. Additionally, leveraging the linear response feature, we explored the X-ray imaging capabilities of our devices, achieving a spatial resolution of 1.0 lp/mm at a 28 cm separation between X-ray source and testing samples and successfully imaging the internal structures of a key handle. Our results demonstrate the feasibility of constructing a perovskite thin-film X-ray detector with superior response performance, reliable reproducibility, and excellent environmental stability on a paper substrate, providing inspiration for further exploration of perovskite film devices for X-ray detection.

## Methods

### Fabrication of paper-based Cs_3_Bi_2_Br_9_ films

The Cs_3_Bi_2_Br_9_ single crystals are fabricated with vertical Bridgman method and the details have been well explained in our previous work^73^. The paper-based Cs_3_Bi_2_Br_9_ film is prepared using an all-dry abrasion deposition method. First, a rectangular mask is created on standard printing paper using tape. Next, a cotton swab is used to pick up the Cs_3_Bi_2_Br_9_ powder and rub it onto the exposed area of the paper until a continuous and uniform film is formed. Finally, the tape is removed, and any remaining powder on the surface is gently blown off with an ear syringe to obtain the paper-based Cs_3_Bi_2_Br_9_ film.

### Fabrication of paper-based Cs3Bi2Br9 X-ray detector

The paper-based Cs_3_Bi_2_Br_9_ X-ray detector adopts a horizontal Au- Cs_3_Bi_2_Br_9_-Au structure. A hard shadow mask is placed over the paper-based Cs_3_Bi_2_Br_9_ film, and a top coplanar gold interdigitated electrode with a thickness of approximately 100 nm is deposited using magnetron sputtering. Three types of devices with electrode finger spacing 1 mm (Type A), 0.5 mm (Type B) and 50 μm (Type C) are fabricated in this work and their geometrical details of the electrode pattern have been illustrated in Fig. S8 and Table S2. For X-ray sensitivity measurements, the X-ray beam was collimated to fully cover the interdigitated region in the devices, ensuring that the active area used for sensitivity calculations precisely matched the illuminated device area (see Fig. S8b). The dose rate is also calibrated using the same method. Current-voltage (*I–V*) and current-time (*I-*t) measurements were performed with a Keithley 6517B source meter. The X-ray tube used was a beryllium window micro-focus X-ray source (LXU-50-50PC1-D) from Megmeet. We determined X-ray detection sensitivity on interdigitated devices (Types A, B, C), with dose-rate ranges: A: 0.7–2.7 mGy/s (Fig. 4b) and 0.4–1.5 mGy/s (Figs. S14, S15); B: 0.9–3.6 mGy/s (Fig. S16); C: 79.6–305.2 μGy/s (Fig. S17). The film resistivity was determined with type A devices (Fig. 4a, Fig. S9). Each dataset is tagged with its device type in the relevant captions.

### Characterization of Cs3Bi2Br9 materials

The morphology and composition of Cs_3_Bi_2_Br_9_ crystals and films were characterized using a field emission scanning electron microscope (ZEISS Sigma 300, Germany) equipped with an energy-dispersive X-ray spectroscopy (EDS) system (Oxford Instruments). UV–visible absorption spectra were measured using an ultraviolet-visible-near-infrared (UV-Vis-NIR) spectrophotometer (Lambda 1050). X-ray diffraction patterns were obtained with an X-ray diffractometer (X’Pert PRO) using Cu K_α_ radiation, covering a range of 5–90° with a step size of 0.0167°. Raman and photoluminescence (PL) spectra were collected using a laser Raman spectrometer (Renishaw inVia Qontor, ATECS Technology Ltd.). X-ray photoelectron spectroscopy (XPS) spectra were acquired using an X-ray photoelectron spectrometer (PHI 5000 Versa Probe III) with Al Kα radiation. Current-voltage (*I–V*) and current-time (*I-T*) measurements were performed with a Keithley 6517B electrometer. The X-ray tube used was a beryllium window micro-focus X-ray source (LXU-50-50PC1-D) from Megmeet. The X-ray imaging system was custom-built in-house by the laboratory and conducted under a tube voltage of 20 kVp, a current of 300 μA (dose rate: 152.8 μGy s^−1^), an operating bias of 100 V and an exposure time = 0.2 s per step. All dark-current measurements were performed under light-shielded conditions to avoid interference from ambient illumination.

## Supplementary information

Supplementary Information

## Data Availability

The datasets generated and/or analyzed during the current study are not publicly available due to their large size and ongoing use in related research but are available from the corresponding author on reasonable request.
