# Supplementary material for: Paper-based lead-free thin film X-ray detectors with high sensitivity and superior environmental stability
Source: Npj Flex Electron. 2026 Jun 13;10(1):105. doi: 10.1038/s41528-026-00607-8 (PMC13623636; doi:10.1038/s41528-026-00607-8)
Supplement: Supplementary file 1 [file 41528_2026_607_MOESM1_ESM.pdf]

Supporting Information: Paper-based lead-free thin film X-ray detectors with high sensitivity and superior environmental stability

Qinghua Zhao[1,2,3,], Yi Liu[1,2,3], Yingying Chen[1,2,3], Wenjie Gong[1,2,3], Xing Li[1,2,3], Boru Zhou[4,5], He Huang[6], Ning Han[7], Shuyu Chen[7], Baoqiang Zhang[8], Jian Li[8], Andres Castellanos-Gomez[9], Volodymyr Gnatyuk[10], Weina Nan[4,5,*], Tao Wang[1,2,3,*]*

Prof. Q. Zhao (corresponding author), Y Liu, Y. Chen, W. Gong, X. Li, Prof. T. Wang (corresponding author)
1 School of Materials Science and Engineering, Northwestern Polytechnical University, Xi'an, 710072, P. R. China
2 State Key Laboratory of Solidification Processing, Northwestern Polytechnical University, Xi'an, 710072, P. R. China
3 Key Laboratory of Radiation Detection Materials and Devices, Ministry of Industry and Information Technology, Xi'an, 710072, P. R. China
E-mail: qinghua_zhao@nwpu.edu.cn; taowang@nwpu.edu.cn

Dr. B. Zhou, Dr. W. Nan (corresponding author)
4 Tianjin Key Laboratory of Functional Crystal Materials, Institute of Functional Crystals, School of Materials Science and Engineering, Tianjin University of Technology, Tianjin 300384, China.
5 State Key Laboratory of Crystal Materials, Tianjin Key Laboratory of Functional Crystal Materials, Institute of Functional Crystals, Tianjin University of Technology, Tianjin 300384, China
E-mail: nwn@email.tjut.edu.cn

H. Huang
6 Digital Publishing Department, Shaanxi Science and Technology Press, Xi'an, 710061, P. R. China

Ning Han, Shuyu Chen
7 Chongqing Jiana Instruments Co, Ltd, Chongqing, 400060, P. R. China

Baoqiang Zhang, Jian Li
8 Shaanxi Imdetek Co., Ltd, Xi'an, 712000, P.R. China

Prof. A. Castellanos-Gomez
9 2D Foundry research group. Instituto de Ciencia de Materiales de Madrid (ICMM-CSIC), Madrid, E-28049, Spain.

Prof. V. Gnatyuk
10 V.E. Lashkaryov Institute of Semiconductor Physics of the National Academy of Sciences of Ukraine, Kyiv 03028, Ukraine.

Keywords: $Cs_3Bi_2Br_9$, perovskite, thin film, paper-based X-ray detector.

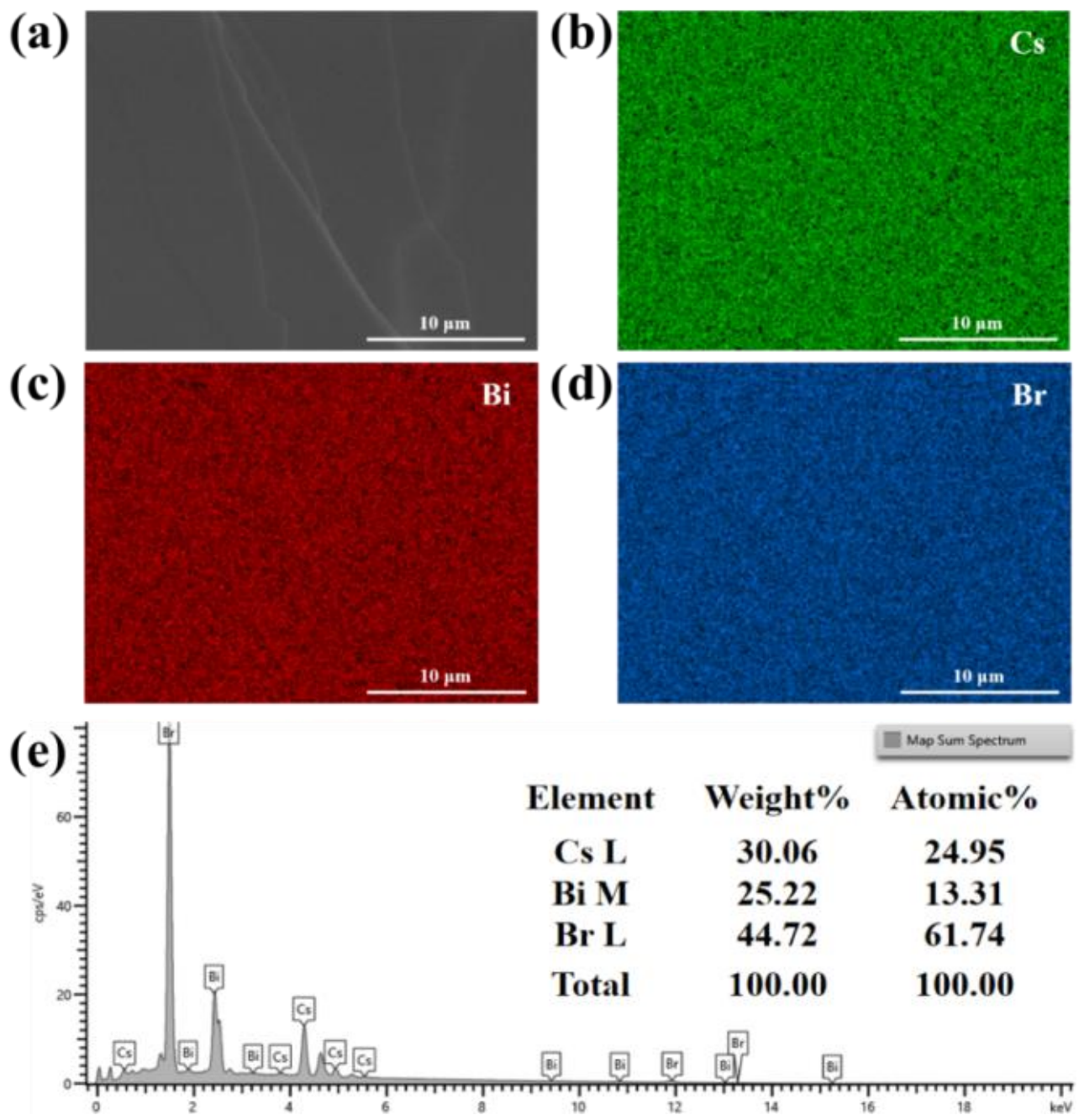

Figure S1. (a) SEM image of the layered structure of a $Cs_3Bi_2Br_9$ single crystal. (b-d) EDS mapping of the Cs, Bi and Br elemental distributions. (e) EDS map summed spectrum result of $Cs_3Bi_2Br_9$ bulk.

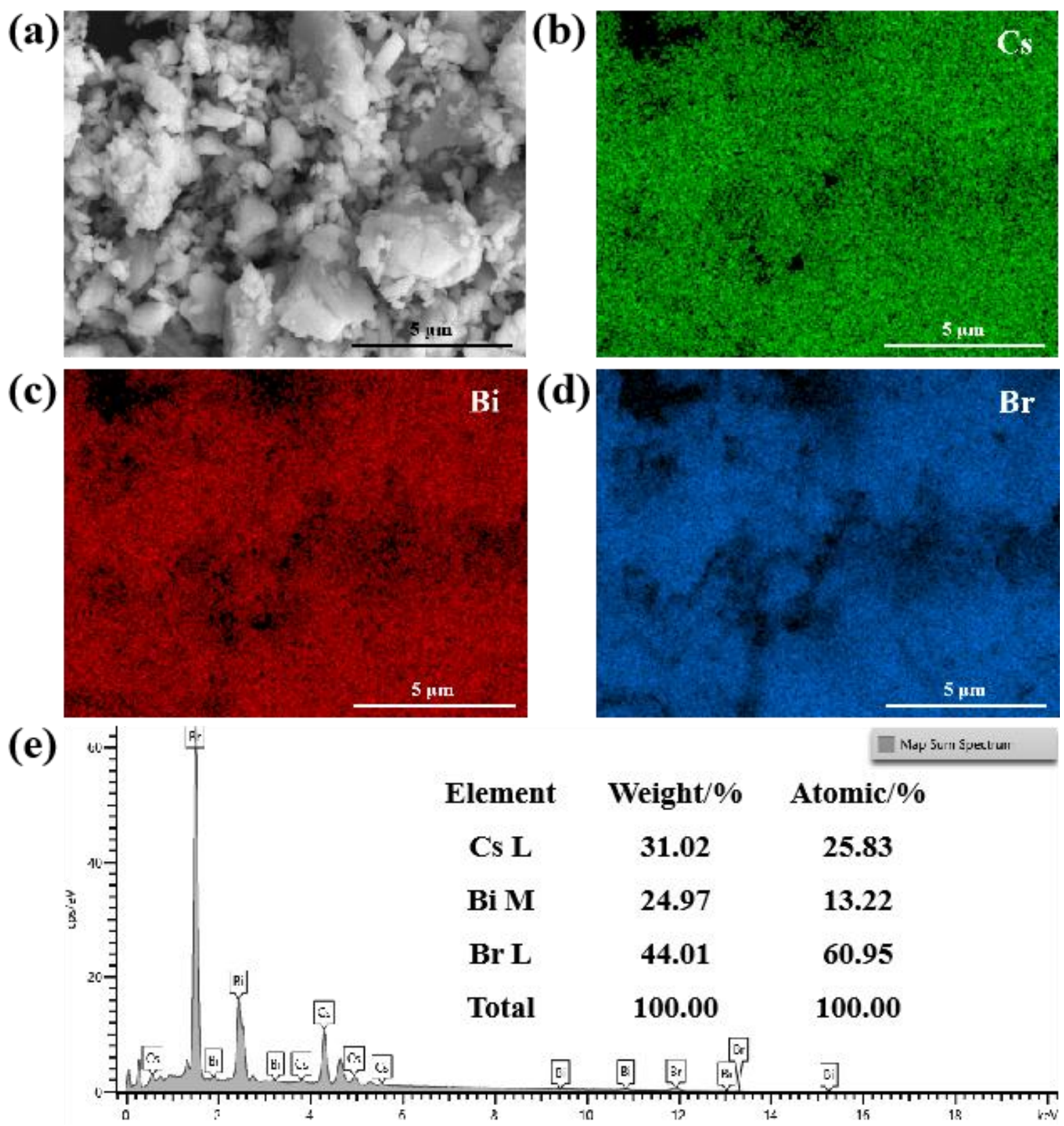

Figure S2. (a) SEM image of $Cs_3Bi_2Br_9$ powder. (b-d) Corresponding EDS mappings of the Cs, Bi and Br elemental distributions. (e) EDS map summed spectrum result of $Cs_3Bi_2Br_9$ powder.

Table S1. Comparison of FWHM values of diffraction peaks between bulk $Cs_3Bi_2Br_9$ and $Cs_3Bi_2Br_9$ Powders

	FWHM values of comparable diffraction peaks			
Material type	(002)	(003)	(005)	(006)
Bulk $Cs_3Bi_2Br_9$	0.0798°	0.1139°	0.091°	0.114°
Powder $Cs_3Bi_2Br_9$	0.114°	0.1587°	0.3192°	0.1938°
Increase (%) relative to bulk	42.9%	39.3%	250.8%	70.0%

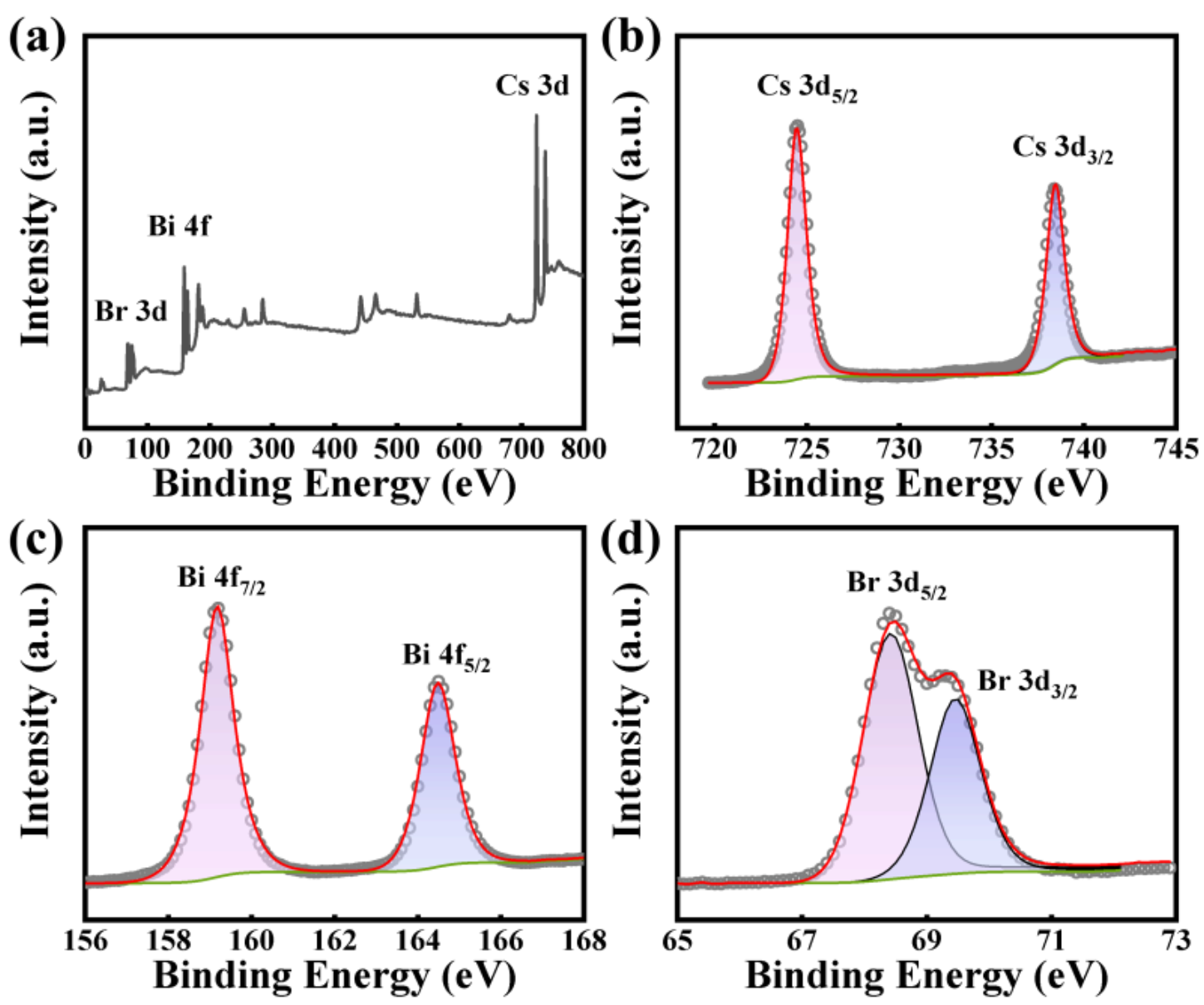

Figure S3. (a) XPS survey spectrum of $Cs_3Bi_2Br_9$ powder. (b-d) Corresponding high-resolution spectra of (b) Cs 3d, (c) Bi 4f, and (d) Br 3d of $Cs_3Bi_2Br_9$ powder.

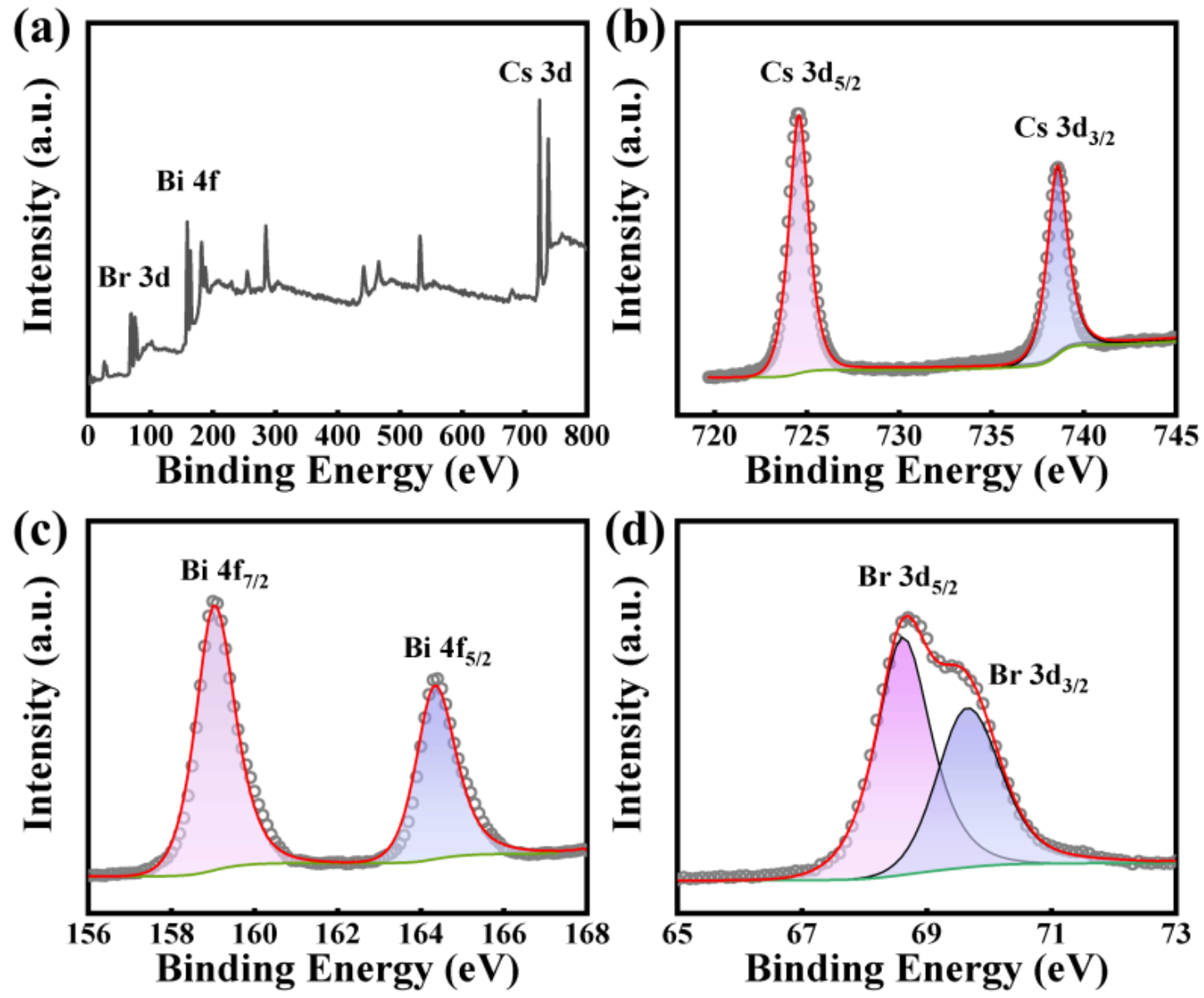

Figure S4. (a) XPS survey spectrum of $Cs_3Bi_2Br_9$ bulk. **(b-d)** Corresponding high-resolution spectra of **(b)** Cs 3d, **(c)** Bi 4f, and **(d)** Br 3d of a $Cs_3Bi_2Br_9$ single crystal.

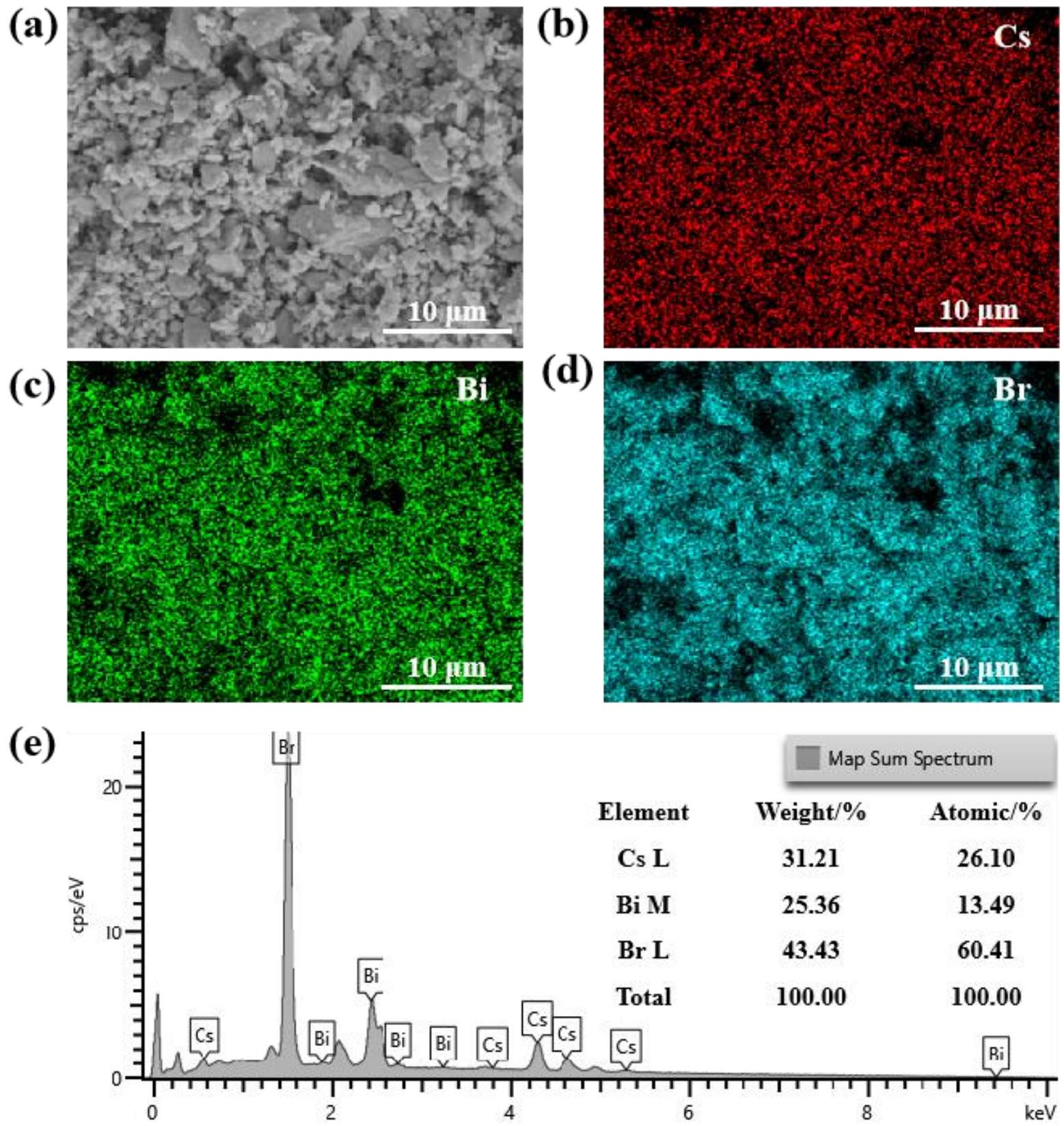

Figure S5. (a) SEM image of the paper-based $Cs_3Bi_2Br_9$ film surface. (b-d) Corresponding EDS mapping of the Cs, Bi and Br elemental distributions. EDS map summed spectrum result of the paper-based $Cs_3Bi_2Br_9$ film surface.

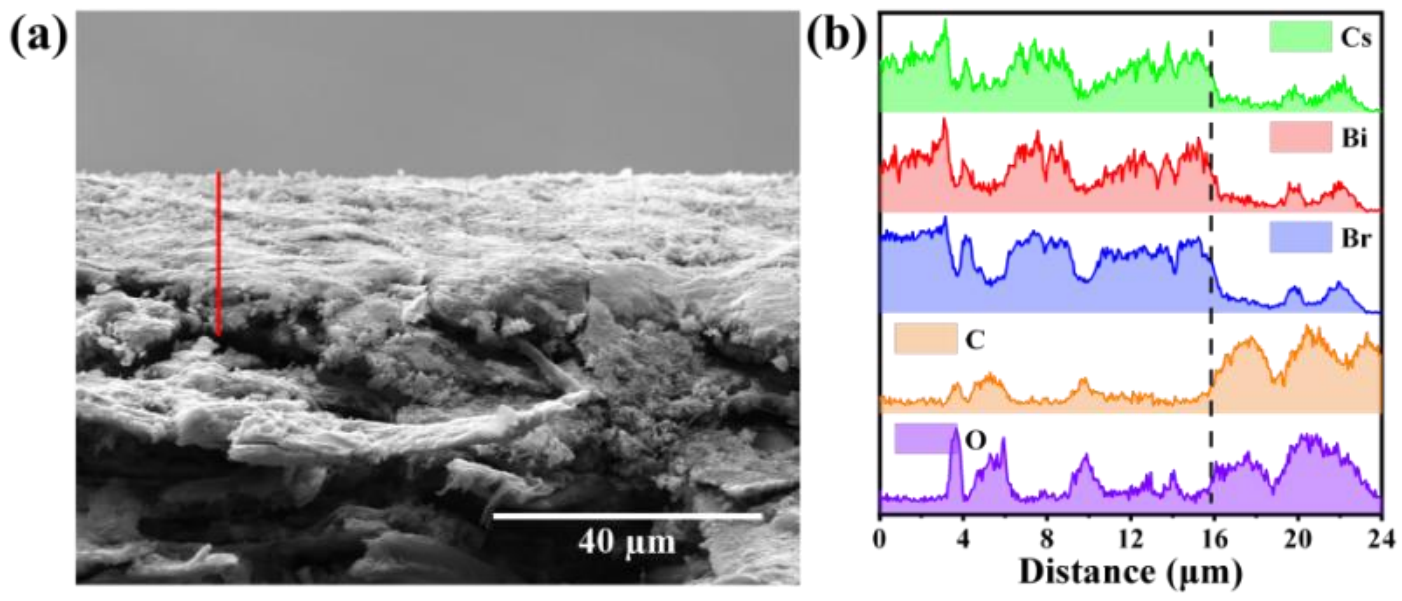

Figure S6. (a) SEM image of paper-based $Cs_3Bi_2Br_9$ film cross-section. (b) EDS compositional scan result along the red line from top to bottom in (a). The sudden change in composition at the dashed line indicates a film thickness of 16 μm. As indicated by the distribution of each element, the boundary is clearly observed, and the thickness can be estimated to be 16 μm, fitting within the fluctuation range.

Supplementary Video 1 shows (i) cotton-swab abrasion of $Cs_3Bi_2Br_9$ powder on office-grade paper, (ii) handling/bending and tape-peel demonstrations confirming that the deposited layer remains adherent during mechanical manipulation. **Supplementary Video 2** presents the electrical verification of the fabricated device.

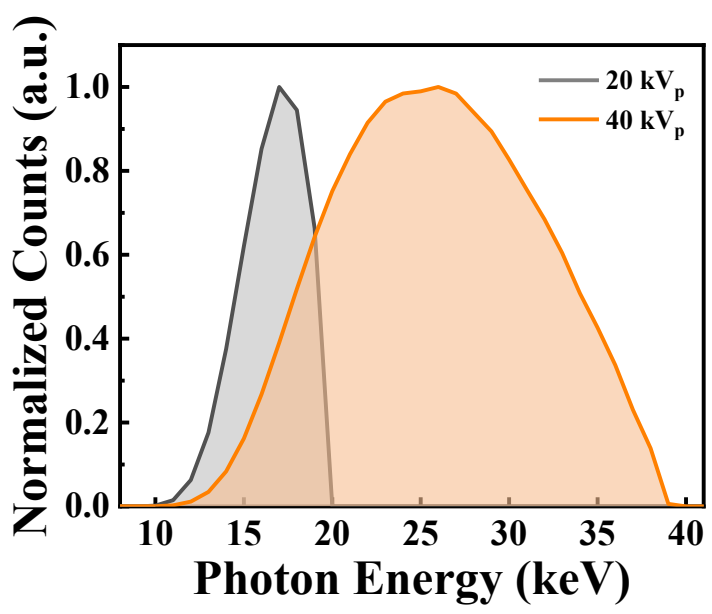

Figure S7. A simulation of photon energy-photon counts at tube voltages of 20 kV$_p$ and 40 kV$_p$. The average energies of X-rays at tube voltages of 20 kV$_p$ and 40 kV$_p$ were calculated to be 16.56 keV and 26.00 keV, respectively.

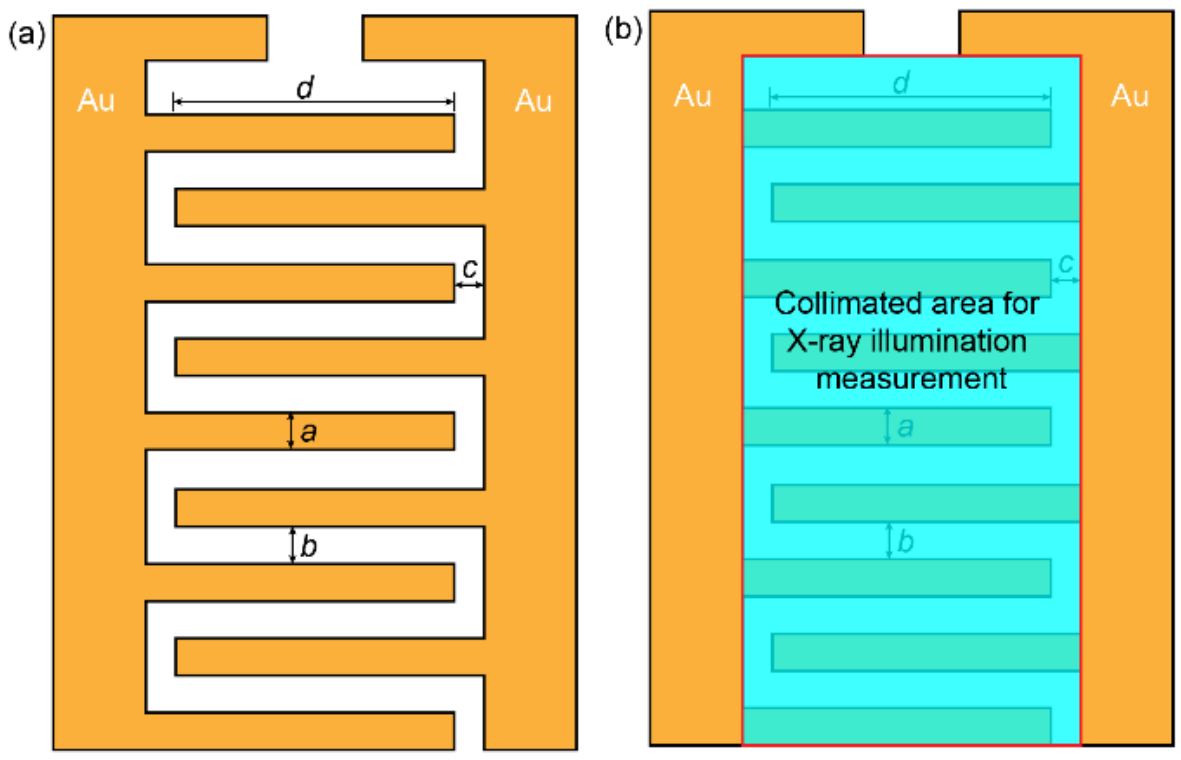

Figure S8. (a) Schematic of the interdigitated Au electrodes on paper-based $Cs_3Bi_2Br_9$ detectors with geometry labels *a* (finger width), *b* (inter-finger gap), *c* (bus-to-bus separation), and *d* (finger length). (b) Collimated X-ray footprint aligned with the interdigitated stripe. The illuminated area for normalization is defined as $A_{illum} = N_{pairs} \times d \times (a + b)$; N_{pairs} is specified for each dataset.

Table S2. Geometrical parameters for three types of paper-based $Cs_3Bi_2Br_9$ devices

Electrode pattern	Finger width *a*	Finger spacing *b*	Electrode gap *c*	Finger length *d*	Electrode pairs N_{pairs}	Area for X-ray illumination $A_{illum} = N_{pairs} \times d \times (a + b)$
A	1 mm	1 mm	1 mm	11-14 mm	8	11×16 mm^2
B	1 mm	0.5 mm	1 mm	9 mm	8	9×12 mm^2
C	50 μm	50 μm	50 μm	1.6 mm	9	1.6×0.9 mm^2

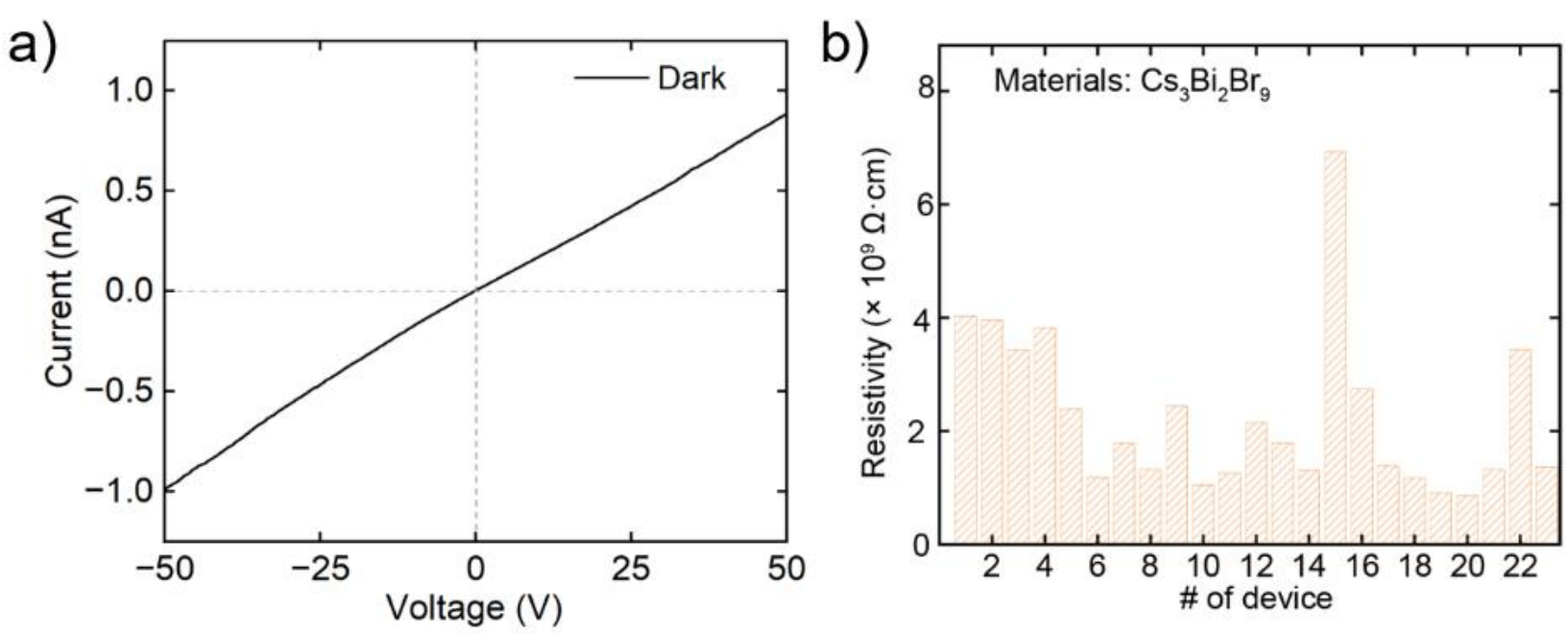

Figure S9. Electrical evidence supporting resistivity extraction.(a) Representative dark I–V curve of a paper-based $Cs_3Bi_2Br_9$ film (size: 5 × 10 mm) showing approximately ohmic

behavior; the device resistance is obtained from linear fitting. (b) Histogram of the resistivity extracted from multiple devices (Gaussian fit shown); inset: distribution of the corresponding resistance values.

Determination of resistivity: For planar interdigitated electrodes, resistivity can be calculated by the following formulas:

$$\frac{1}{R'} = \frac{4}{\frac{b}{c}ln(\frac{2c}{d-c}+1)R_f} + \frac{4}{\frac{c}{b}ln(\frac{2b}{a}+1)R_f} \tag{1}$$

$$\frac{1}{R} = \frac{n}{R'} \tag{2}$$

$$R_f = \frac{\rho}{\delta} \tag{3}$$

where R' is the distributed resistance per period, R_f is the sheet resistance, R is the total resistance of the thin film, n is the number of distributed resistance periods, ρ is the total resistivity of the thin film, δ is the film thickness, and a, b, c, and d represent the finger width, finger spacing, electrode gap, and finger length, respectively.

For the device discussed in the main text (Figure 4a), the parameters are fitted as: n = 3.5, a = b = c = 1 mm, and d = 14 mm. By fitting the dark-field I–V curve, the total film resistance was determined to be 5.45×10^{10} Ω. Substituting these values into the above equations yields a sheet resistance $R_f = 4.88 \times 10^{12}$ Ω, and consequently, the film resistivity $\rho = 6.90 \times 10^9$ Ω·cm.

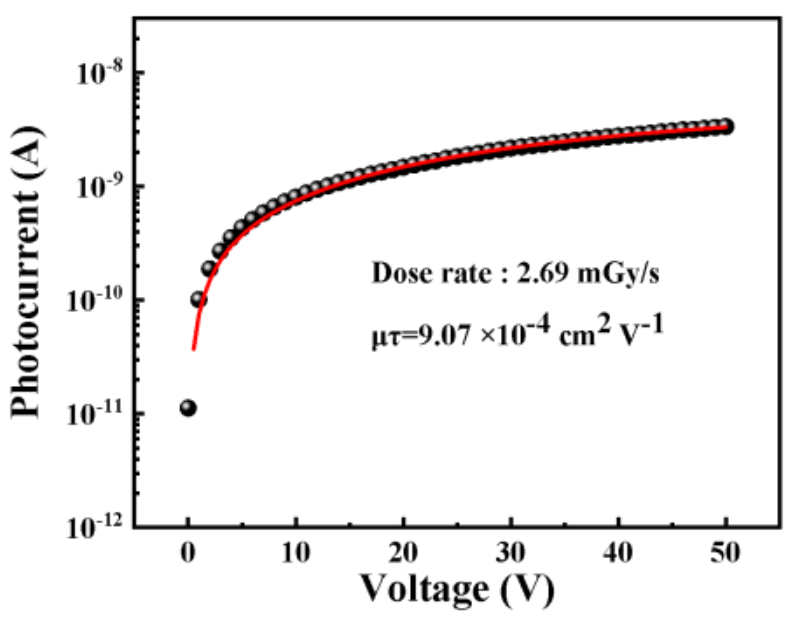

Figure S10. Current-voltage curve under X-ray irradiation with a dose rate of 2.69 mGy·s^{-1}. The $\mu\tau$ of 9.07 × 10^{-4} cm^2·V^{-1} was obtained from the fitting the following hecht equation[52]: $I = I_0 \frac{\mu\tau V}{d^2}(1 - exp(-\frac{d^2}{\mu\tau V}))$

where I_0 is the saturation photocurrent, V is the applied voltage, d is the channel distance, μ is the carrier mobility, and τ is the carrier lifetime.

Compared with bulk $Cs_3Bi_2Br_9$ single crystals, the resistivity measured from our paper-based abrasion-deposited thin-film devices (6.9 × 10^9 Ω·cm) is ~two orders of magnitude lower. This difference likely originates from several microstructural and interfacial factors: (1) Grain boundaries and intergranular pathways. The abrasion-deposited layer consists of loosely packed microcrystals/flakes with abundant boundaries, which can introduce defect-rich regions and facilitate leakage/hopping conduction. (2) Porous cellulose interface. The rough and porous paper matrix forms a composite interface with local variations in coverage and electric-field distribution, which may create low-resistance pathways and enhance surface/interfacial conduction. (3) Ambient moisture–assisted conduction. Under typical ambient humidity (~40–45% RH), moisture adsorption by cellulose can increase surface/ionic conduction along the paper–perovskite interface, reducing the effective resistance. (4) Coplanar interdigitated geometry effects. In planar interdigitated devices, fringing fields and contact nonuniformity can further make the extracted value reflect an effective resistivity rather than the intrinsic bulk value of a dense single crystal. (5) oxygen/water adsorption and minor surface Bi–O formation. Powder-derived surfaces have a much higher specific surface area and are more susceptible to oxygen/water adsorption and minor surface Bi–O formation. Collectively,

these effects can lower the effective resistivity of paper-based thin-film devices compared with densely packed, defect-free bulk single crystals.

In addition to resistivity, the μτ product reported in this work is obtained from field-dependent photocurrent analysis in a coplanar interdigitated configuration and therefore should be interpreted as an effective μτ under our device geometry and operating field. μτ values reported for bulk single-crystal detectors are often extracted using different geometries, thicknesses, contact configurations, and analysis methods; hence small numerical differences (e.g., 9.07×10^{-4} vs 8.32×10^{-4} $cm^2{\cdot}V^{-1}$) are within the expected uncertainty and do not necessarily indicate intrinsically superior bulk transport in the thin film.

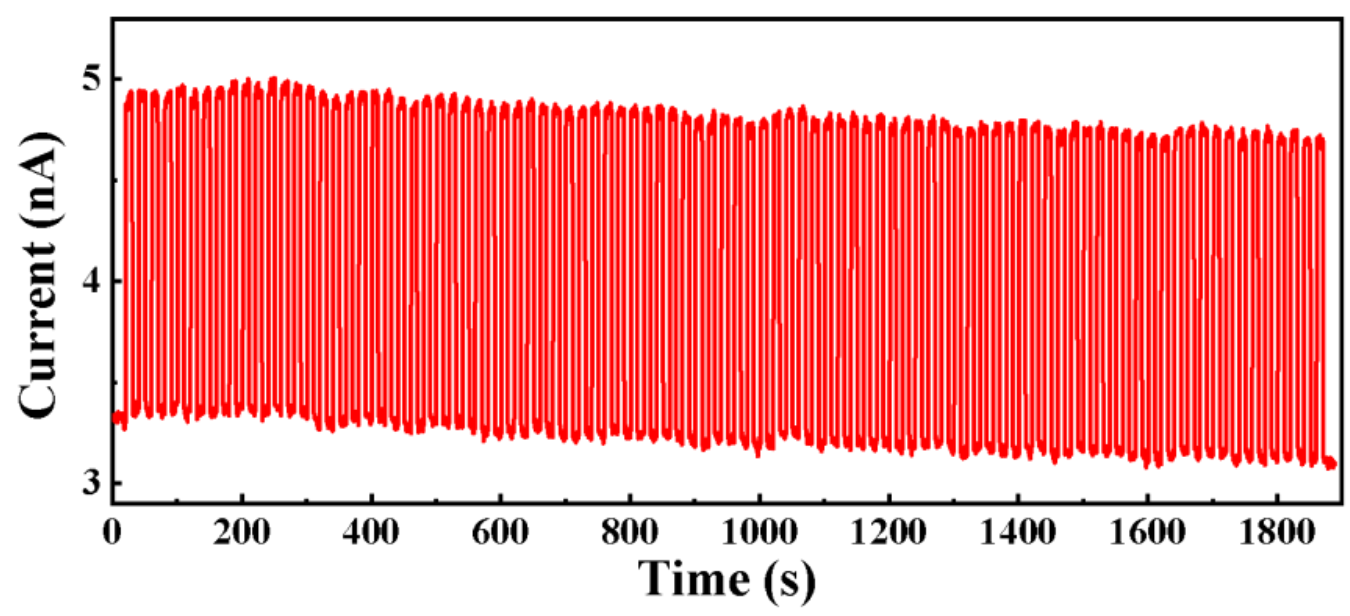

Figure S11. Current-time curve of the X-ray response within 1800 seconds of continuous operation. Within ~100 cycles, the net photocurrent remains essentially unchanged, although both the dark current and photocurrent decrease slightly. This indicates that the device has excellent operational stability. (the device electrode follows a Type C layout)

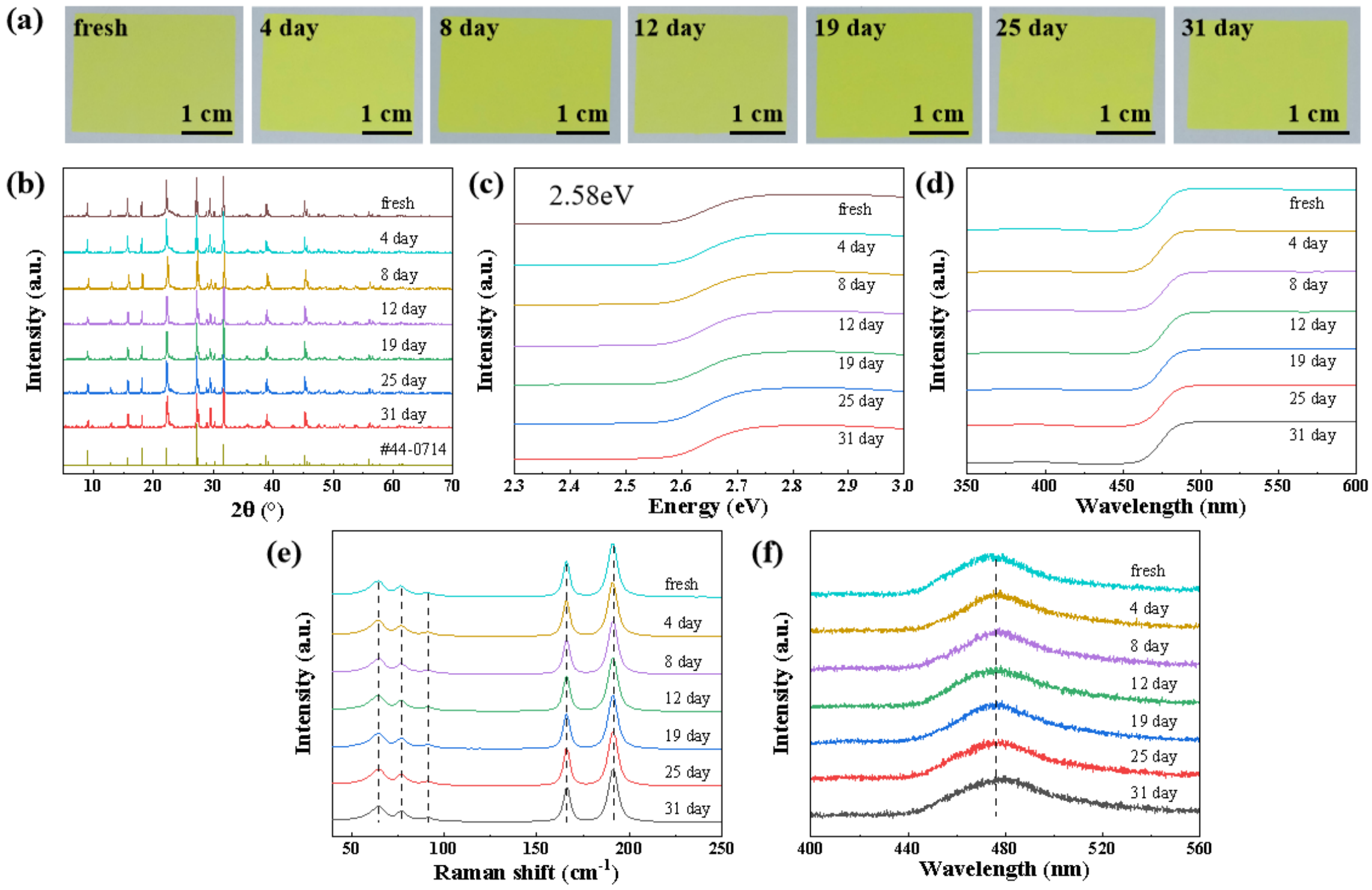

Figure S12. (a) Comparison of optical images, (b) XRD patterns, (c) UV absorption, (d) UV transmission, (e) Raman, and (f) PL spectra of $Cs_3Bi_2Br_9$ films placed under ambient condition for 0, 4, 8, 12, 19, 25, and 31 days.

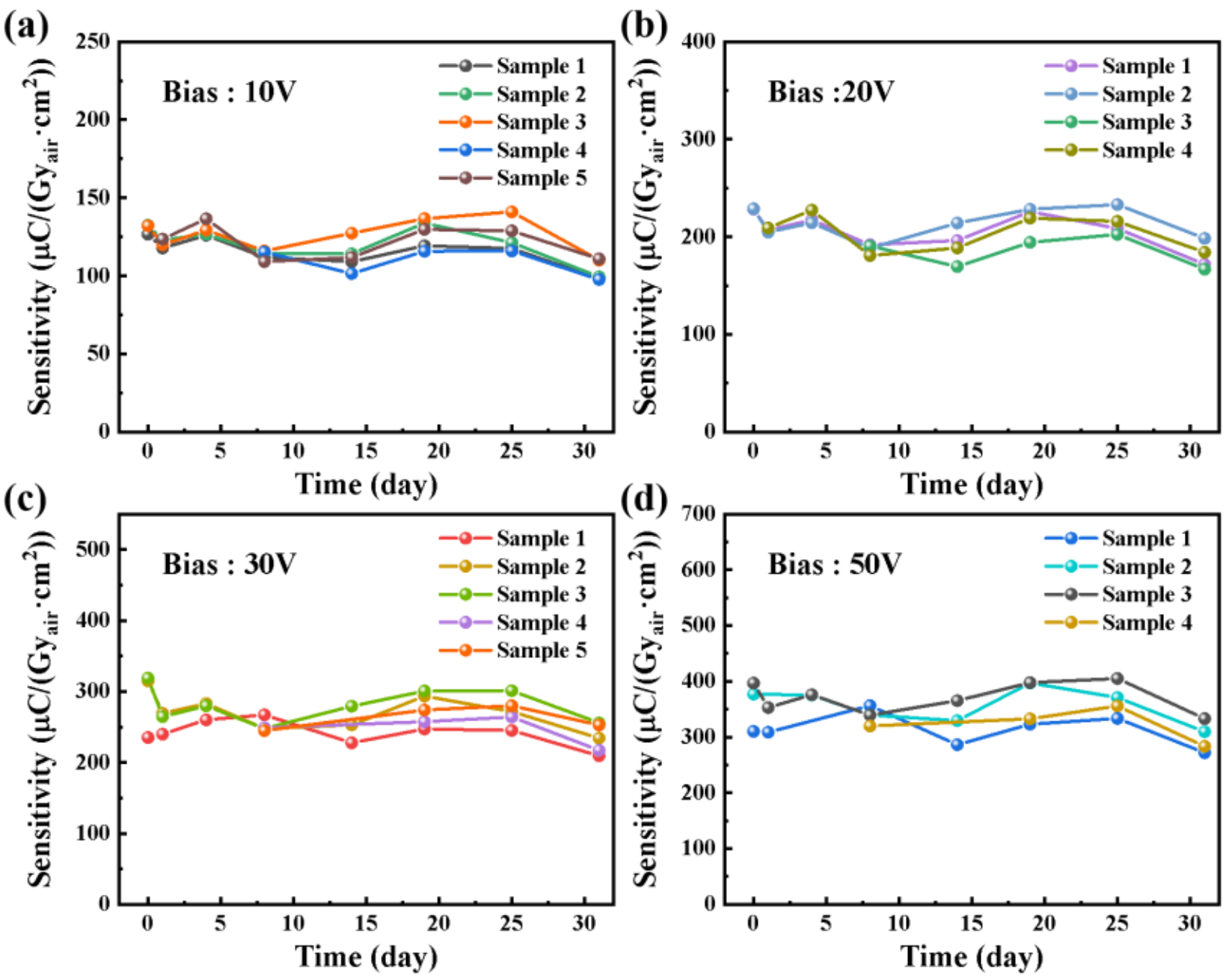

Figure S13. Sensitivity of $Cs_3Bi_2Br_9$ devices placed under ambient conditions for 0, 1, 4, 8, 14, 19, 25, and 31 days at (a) 10 V, (b) 20 V, (c) 30 V, and (d) 50 V bias. (the device electrode follows a Type C layout)

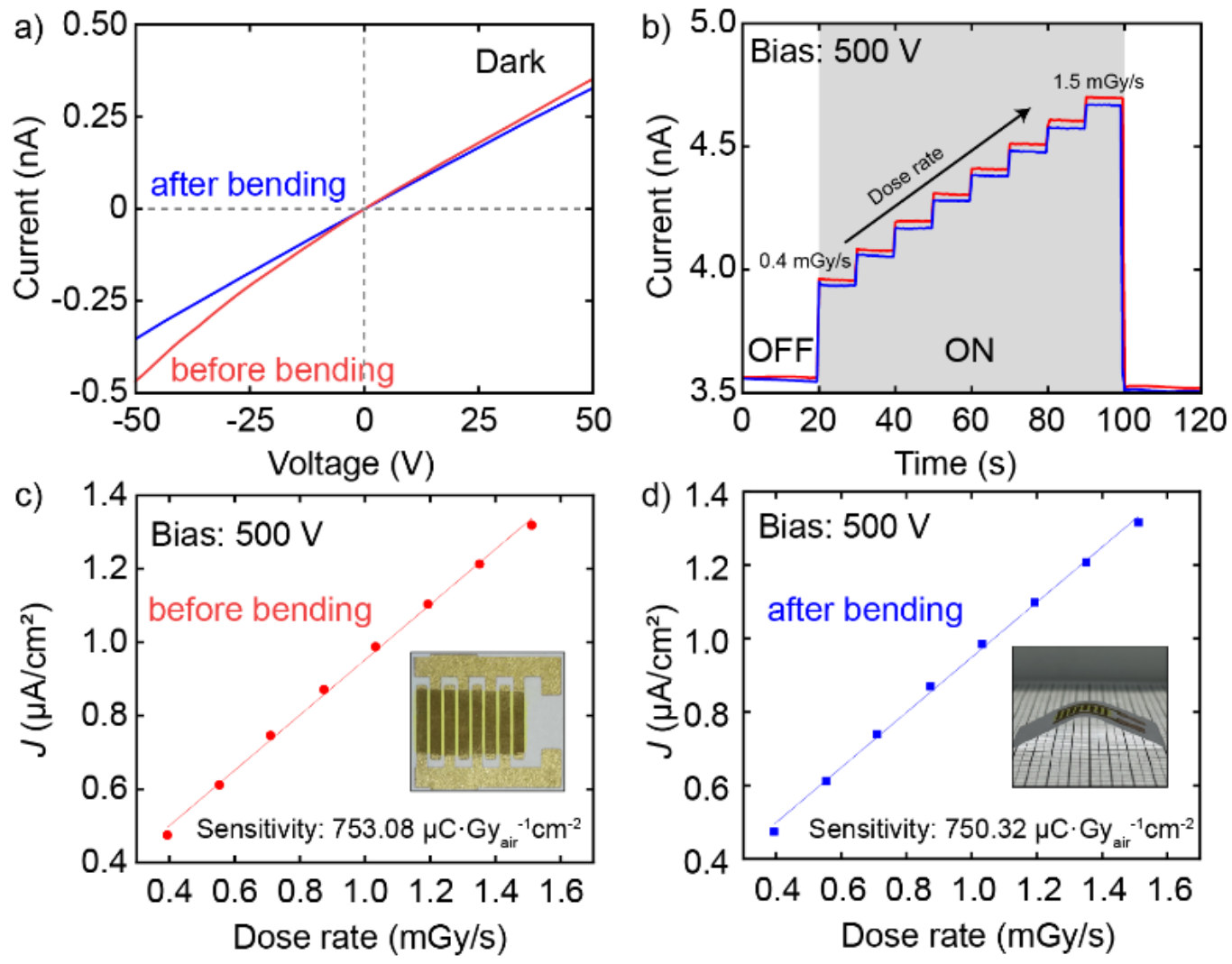

Figure S14. Effect of mechanical bending on the X-ray detection performance of a paper-based $Cs_3Bi_2Br_9$ radiation detector (the device electrode follows a Type A layout). (a) Current–voltage (*I*–*V*) characteristics measured before and after 100 bending cycles. (b) Time-dependent current (*I*–t) response under X-ray irradiation before and after bending. (c, d) X-ray detection sensitivity of the device measured before (c) and after (d) 100 bending cycles.

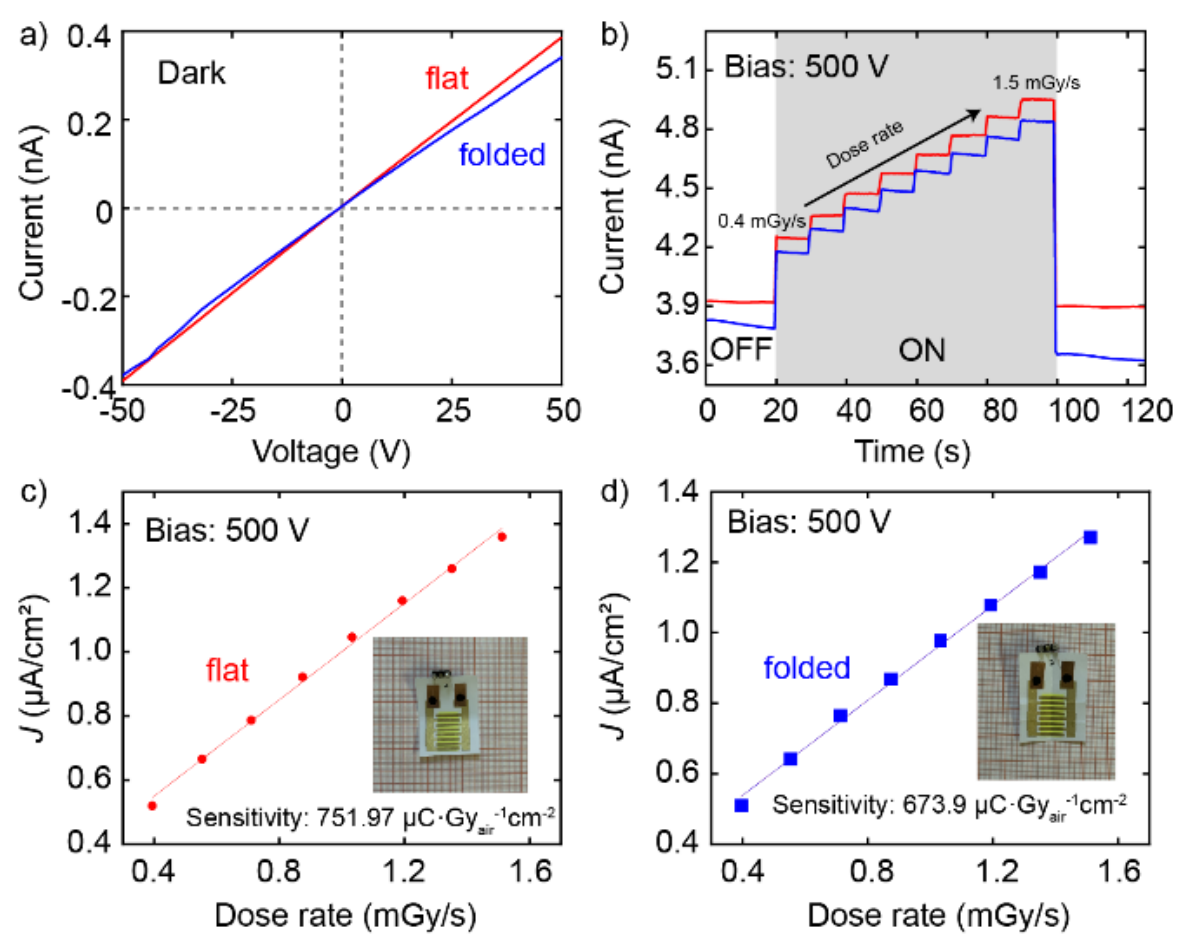

Figure S15. Effect of static folding on the X-ray detection performance of a paper-based $Cs_3Bi_2Br_9$ radiation detector (the device electrode follows a Type A layout). (a) Dark *I*–*V* characteristics of the device measured in the flat (red) and folded (blue) states, showing minimal variation in leakage current. (b) Time-dependent current response (*I*–t) under X-ray irradiation at a bias of 500 V and dose rates ranging from 0.4 to 1.5 mGy/s, recorded for both flat and folded configurations. (c, d) X-ray detection sensitivity as a function of dose rate under 500 V bias for the device in flat (c) and folded (d) states. The sensitivity decreased from 751.97 to 673.9 $\mu C{\cdot}Gy_{air}^{-1}{\cdot}cm^{-2}$ after folding, indicating reasonable retention of detection performance.

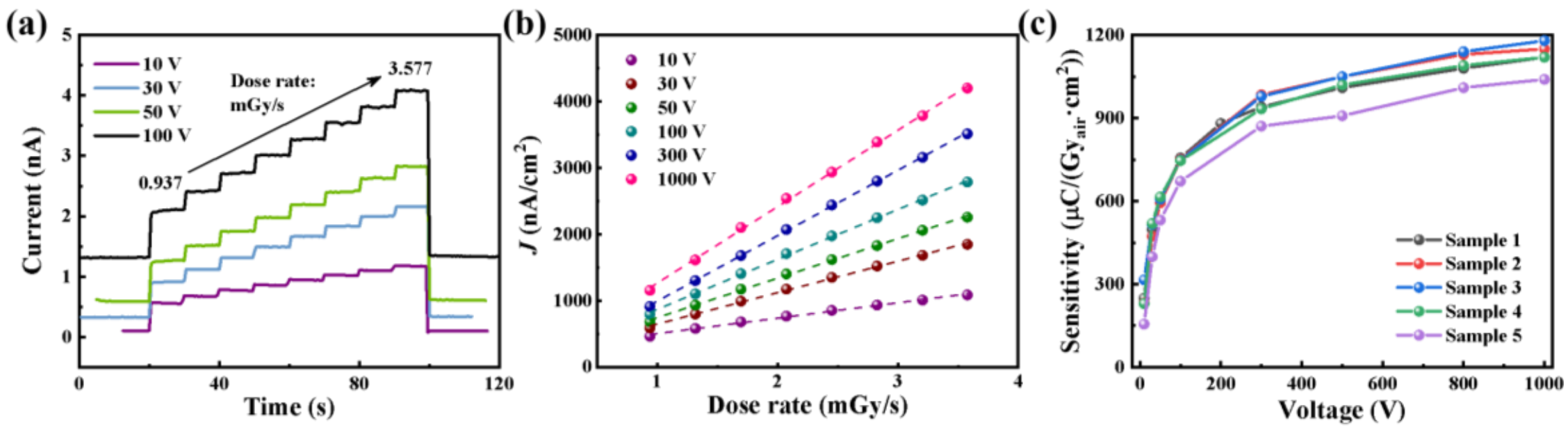

Figure S16. X-ray detection properties of $Cs_3Bi_2Br_9$ devices with a channel distance of 0.5 mm (the device electrode follows a Type B layout). (a) Current-time curves at *V* = 10 V (purple), 30 V (blue), 50 V (green), and 100 V (black) with varying the radiation doses rates from 0.9 $mGy{\cdot}s^{-1}$ to 3.6 $mGy{\cdot}s^{-1}$. (b) Photocurrent density as a function of X-ray illumination dose rate recorded at six different bias voltages. (c) Variation of X-ray detection sensitivity plotted as a function of applied bias electric field.

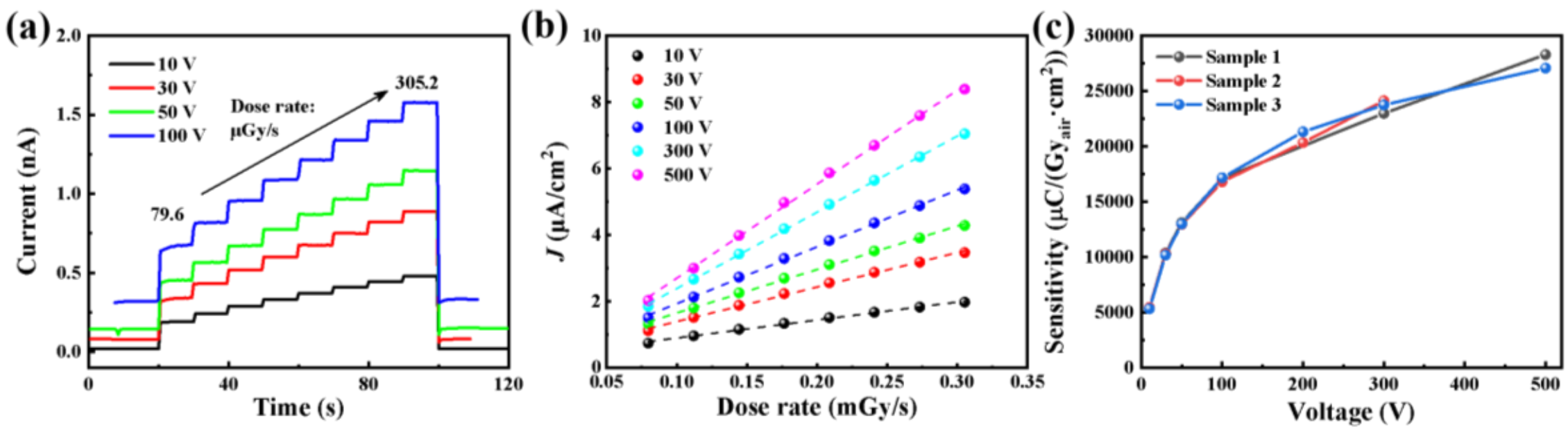

Figure S17. X-ray detection properties of $Cs_3Bi_2Br_9$ devices with a channel distance of 50 μm (the device electrode follows a Type C layout). (a) Current-time curves at V = 10 V (black), 30 V (red), 50 V (green), and 100 V (blue) with varying radiation doses rates from 79.6 μGy·s^{-1} to 305.2 μGy·s^{-1}. (b) Photocurrent density as a function of X-ray radiation dose rate recorded at six different bias voltages. (c) Variation of X-ray detection sensitivity plotted as a function of applied bias electric fields.

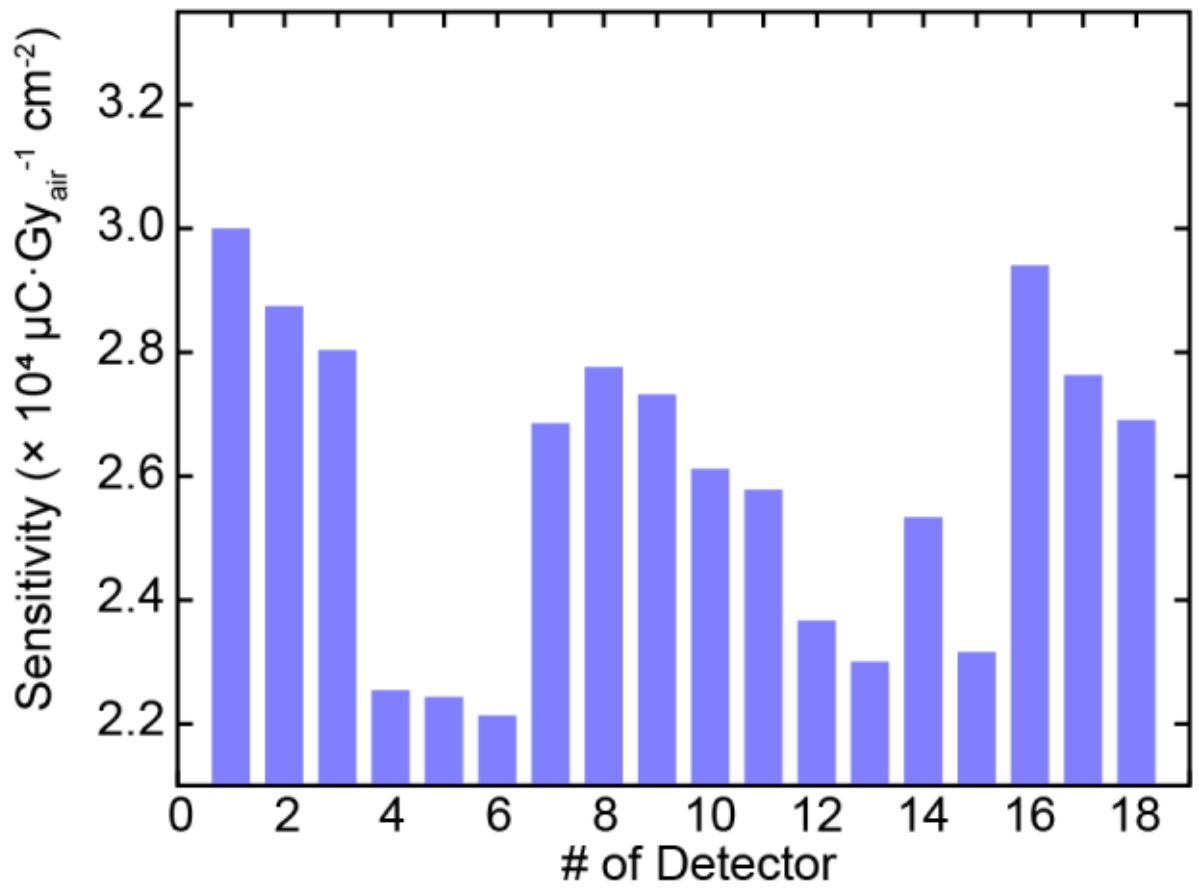

Figure S18 Statistical analysis of sensitivity collected from 18 $Cs_3Bi_2Br_9$ devices with a channel distance of 50 μm (the device electrode follows a Type C layout).

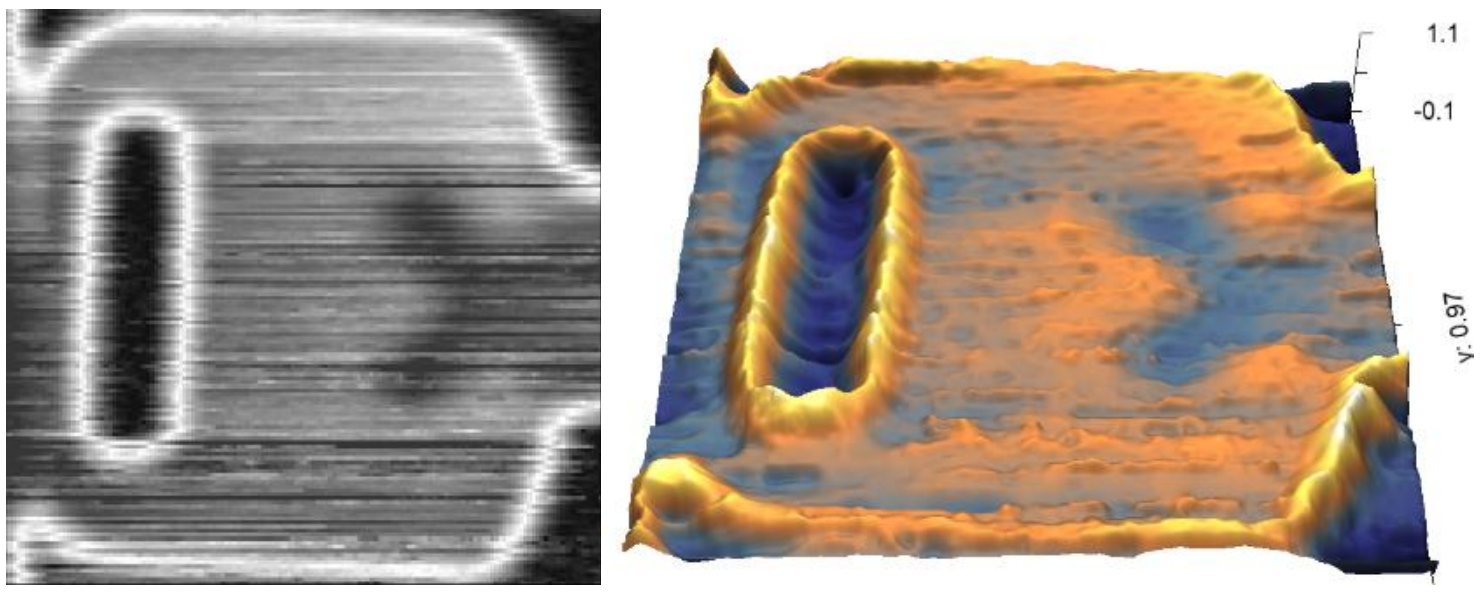

Figure S19. X-ray images of a key handle plotted in gray scale and 3D representation.

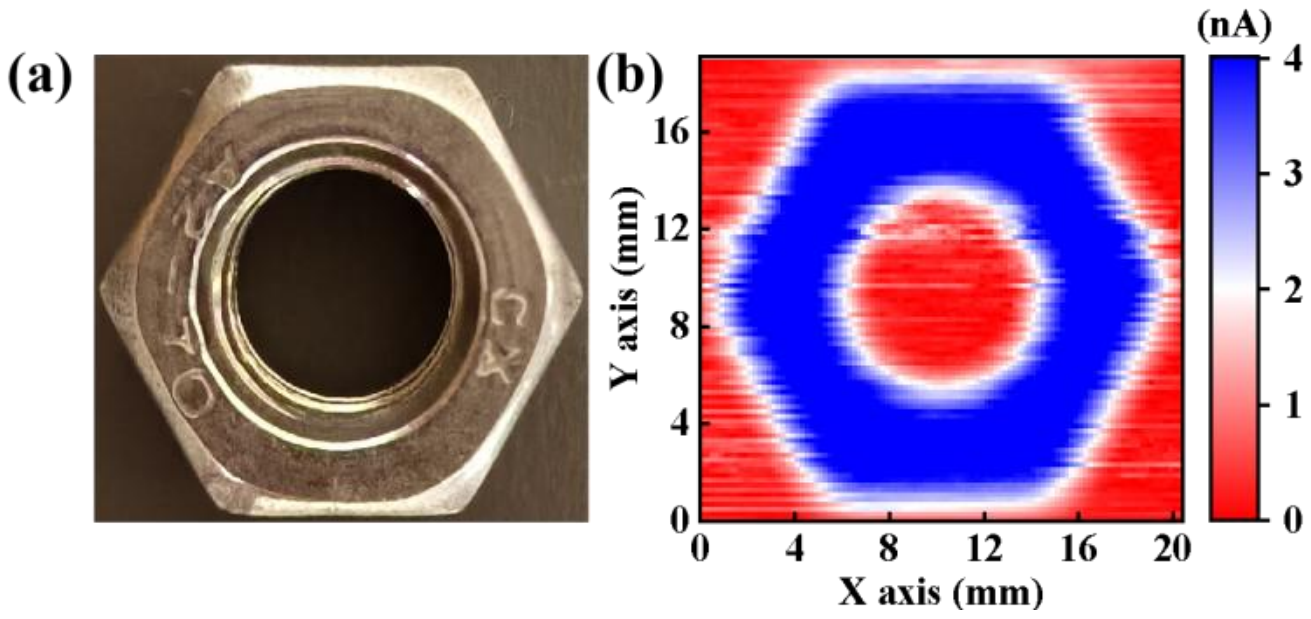

Figure S20. (a) Optical image of the hexagonal nut and (b) the corresponding X-ray imaging result.

Table S3. Comparison of the figure-of-merits between our device and X-Ray detectors reported in literature.

Materials	E_{ph} (keV), V_p (kV_p)	thickness (mm)	μτ product ($cm^{-2}V^{-1}$)	Sensitivity ($\mu C \cdot Gy^{-1} cm^{-2}$)	LOD ($nGy \cdot s^{-1}$)	Resistivity (Ω·cm)	Ref.
α-Se	13-110 keV	-	~10^{-7}	20@10^4 V·mm^{-1}	1-10 μGy	-	-
Si	1-60 keV	-	10^{-4}-10^{-6}	0.02-1	0.1	10^3-10^4	-
CdTe	0-100 keV	-	10^{-4}-10^{-6}	-	-	10^8-10^9	-
CZT	10 keV–6 MeV	-	>10^{-3}	-	-	~10^{10}	-
$Cs_3Bi_2I_9$	-	8×10^{-5}	-	127.23	7400	-	NPG Asia Materials 2024, 16 (1), 34.
Cs_2TeI_6	20 kV_p	0.003	-	76.27	-	~10^{10}	ACS Applied Materials & Interfaces 2021, 13 (20), 23928-23935.
$Cs_2AgBiBr_6$ film	-	0.1	-	40 (400 V)	-	~10^{11}	*Journal of Materials Chemistry C 2018, 6 (44), 11961-11967.*
$(BA)_2Cs_9Ag_5Bi_5Br_{31}$	-	-	-	~47 (400 V/mm)	138	~10^{10}	RSC Adv., 2024, 14, 19124
$Cs_2AgBiBr_6$ (single crystal)	50 keV	-	-	316	59.7	5.5×10^{11}	Advanced Materials, 2018, 30(46): e1804450

$CsPbBr_3$	120 keV	-	8.11×10^{-4}	46180	10.81	1.61×10^{10}	Nature Photonics volume 18, pages870–877 (2024)
$Cs_3Bi_2I_9$	40 kV_p	1.2	7.97×10^{-4}	1652.3	130	2.79×10^{10}	Nat. Commun., 2020, 11: 2304
$MA_3Bi_2I_9$	100 kV_p	2.5	2.8×10^{-3}	10620	0.62	5.27×10^{11}	J.Energy Chem., 2020, 49: 299
$(F\text{-}PEA)_2PbI_4$	120 keV	1.5	5.1×10^{-4}	3402	23	1.36×10^{12}	Adv. Mater., 2020,32(40): 2003790
$BDAPbI_4$	40 kV_p	-	4.43×10^{-4}	242	430	-	Angew. Chem. Int. Ed., 2020, 59(35): 14896
$Rb_3Bi_2I_9$	30 keV	1	2.51×10^{-3}	159.7	8.32	2.3×10^{9}	Adv. Funct. Mater., 2020, 30(24): 1910648
$CsPbI_3$	50 kV_p	0.25	3.63×10^{-3}	2370	219	7.4×10^{9}	J. Phys. Chem. Lett. 11, 432-437 (2020)
$MAPbBr_3$	120 keV	4	2.1×10^{-2}	1274	560	5.4×10^{8}	Sci. Bull. 66, 2199-2206 (2021)
$Cs_3Bi_2I_8Br$	100 keV	2.5	-	13000	28.6	1.99×10^{10}	Angew. Chem. Int. Ed. 62, e202315817 (2023)
$(PMA)_2PbI_4$	40 kV_p	0.9	8.05×10^{-3}	283	2130	-	Appl. Phys. Lett., 2022, 120(1): 011901
$(BDA)CsPb_2Br_7$	50 kV_p	0.7	2.33×10^{-5}	725.5	3810	4.35×10^{10}	J. Phys. Chem. Lett.,2022, 13(5): 1187
BA_2PbBr_4	50 keV	0.16	1.1×10^{-5}	726.18	0.0082	8.51×10^{11}	Energy Environ. Mater. (2022)
$Cs_3Bi_2Br_9$	40 kV_p	0.014	9.07×10^{-5}	(2-3) $\times10^{4}$	~74	6.93×10^{9}	This work
